# Sleep duration and risk of cardio-cerebrovascular disease: A dose-response meta-analysis of cohort studies comprising 3.8 million participants

**DOI:** 10.3389/fcvm.2022.907990

**Published:** 2022-09-27

**Authors:** Yi-Ming Huang, Wei Xia, Yi-Jun Ge, Jia-Hui Hou, Lan Tan, Wei Xu, Chen-Chen Tan

**Affiliations:** ^1^Department of Neurology, Qingdao Municipal Hospital, Qingdao University, Qingdao, China; ^2^Department of Cardiology, Qingdao Municipal Hospital, Qingdao University, Qingdao, China

**Keywords:** sleep duration, cardiovascular disease, cerebrovascular disease, meta-analysis, dose-response

## Abstract

**Background:**

The effect of extreme sleep duration on the risk of cardiovascular and cerebrovascular diseases (CCDs) remains debatable. The pathology of CCDs is consistent in some respects (e.g., vascular factors), suggesting that there may be an overlapping range of sleep duration associated with a low risk of both diseases We aimed to quantify the dose-response relationship between sleep duration and CCDs.

**Study objective:**

To explore whether there is an optimal sleep duration (SD) in reducing the risk of CCDs.

**Methods:**

PubMed and EMBASE were searched until June 24, 2022 to include cohort studies that investigated the longitudinal relationships of SD with incident CCDs, including stroke and coronary heart disease (CHD). The robusterror meta-regression model (REMR model) was conducted to depict the dose-response relationships based on multivariate-adjusted risk estimates.

**Results:**

A total of 71 cohorts with 3.8 million participants were included for meta-analysis, including 57 for cardiovascular diseases (CVD) and 29 for cerebrovascular disease. A significant U-shaped relationship was revealed of nighttime sleep duration with either cardiovascular or cerebrovascular disease. The nighttime sleep duration associated with a lower risk of CVD was situated within 4.3–10.3 h, with the risk hitting bottom at roughly 7.5 h per night (*p*_*non–linearity*_ < 0.0001). Sleep duration associated with a lower risk of cerebrovascular diseases ranges from 5 to 9.7 h per night, with the inflection at 7.5 h per night (*p*_*non–linearity*_ = 0.05). Similar non-linear relationship exited in daily sleep duration and CCDs. Other subgroup analyses showed non-linear relationships close to the above results.

**Conclusion:**

Rational sleep duration (7.5 h/night) is associated with a reduced risk of cardio-cerebrovascular disease for adults.

## Introduction

Ischemic cardiovascular disease contributes significantly to global morbidity and mortality, with approximately 18 million deaths per year, nearly 9 million because of coronary heart disease (CHD) and 6 million as a result of stroke (1, 2). Also, 15 million experience a stroke annually. Concerning the CVD-related deaths in most advanced economies, more than half occur in the middle-aged and one-third in the elderly population (3). Variations in the pathological mechanisms, regional susceptibilities, and stage failures are present in cardiovascular and cerebrovascular diseases (CCDs).

Moreover, the modifiable risk factors that influence the occurrence of CVD encompass stress, depression and anxiety, cholesterol and lipids, unhealthy diet, obesity, physical inactivity, diabetes mellitus, smoking, and hypertension. In this case, lifestyle changes and medications are crucial for the prevention and management of CVD. However, the latter approach could also have negative consequences, such as experiencing a greater degree of side effects, worsening compliance rates, and increasing burden on the organs due to the medications. Such drawbacks underscore the importance of pursuing the former option instead (a healthy lifestyle), which includes enhancing the quality of one’s sleep.

The existing literature has underscored how addressing sleep disorders could serve as a promising goal in preventing CCDs, but the public’s awareness of its importance is yet to be developed, and the evidence is still limited. A key indicator of sleep quality is the duration, where sleep deprivation or oversleeping are linked to the increased risk of CCDs, but since this relationship was found to have a null, J-, or U-shaped curve, it remained controversial. In specific areas, e.g., vascular factors, the pathology of CCDs is reliable, implying the existence of a shared range of sleep deprivation that could decrease people’s risks of both diseases. A limited number of studies examined the overlapping sleep duration for a lower risk of acquiring them. While new papers have been published on this subject in the past 5 years, the findings were inconsistent (4–12). Thus, through meta-analysis, this research seeks to revise the association of sleep duration with CCDs and unveil the sleep duration range valuable to preventing these diseases.

## Materials and methods

### Literature search strategy and selection criteria

We conducted a literature search on PubMed (Medline) and EMBASE to obtain cohort studies till June 2022, using the following unrestricted search terms: cohort, longitudinal, prospective, retrospective, nested case-control, stroke, cerebral hemorrhage, cerebral infarction, cerebrovascular accident, CVD, cardiovascular disease, CHD, myocardial infarction, CHD, MI, sleep duration, for the search terms see Supplementary File. In addition, we reviewed studies included in the previously published Meta-analysis to identify additional relevant studies. Studies were accepted for inclusion if they also met the following criteria: (1) it is a longitudinal study; (2) the study explored the association between daily or nighttime sleep duration with incident risk of cardiovascular disease and cerebrovascular disease; (3) relative risk (RR) with a 95%confidence interval (CI) was reported for at least two categories of sleep duration. Studies will be excluded if they fail to meet the abovementioned inclusion criteria. We included a larger sample size or longer follow-up if the study population was reported repetitively.

### Data extraction

Two researchers (H-YM and T-CC) independently extracted data using a standardized electronic format. The following information was extracted from each study, including the first author, publication year, study name, country, follow-up duration, age, gender, the sample size for analysis, number of incident cases, sleep duration, diagnosis method for cardio-cerebrovascular diseases, adjusted confounders, and the multivariable-adjusted risk estimates. The results by gender were treated as two separate reports.

### Assessment of the study quality

A modified Newcastle-Ottawa Quality Assessment Scale (mNOS) (13, 14) was used to assess the quality of qualified studies. The total score of mNOS was regarded here as a proxy to determine the overall risk of bias for every single investigation. The score for each item evaluated the associated risk of bias (Supplementary Table 1) in three domains: selection (generalizability, assessment bias, and potential reverse causality), confounding bias, and outcome (assessment bias and attrition bias).

### Statistical analysis

The multivariable–adjusted risk estimates and 95% CI were log-transformed and pooled using random models (DerSimonian-Laird method). Some studies reported odds ratios (OR) but not relative risk (RR) or HRs. Since ORs tend to overestimate the effect size compared with RRs/HRs, especially when the incidence is not low, we used the following algorithm to convert ORs to RRs: (15).


$${\text{RR}}_{adjusted}={\text{OR}}_{adjusted}/[\left(\text{1}-{\text{P}}_{0}\right)+({\text{P}}_{0}\times {\text{OR}}_{adjusted}]$$

P_0_ indicates the incidence of endpoint (cardiovascular or cerebrovascular diseases) in the non-exposed group of cohorts. When P_0_ is not available, the incidence rate of the total sample was used as a proxy (15).

Analyses were performed separately according to outcome (CCDs) and exposure (nighttime and daily sleep duration). We used the inverse variance weighted least squares regression with cluster robust error meta-regression model (REMR model) (16, 17). Heterogeneity was assessed by *Q*-test and quantified by the *I*^2^ metric. Subgroup analyses by gender and outcome group were performed. The robustness of the results was examined by excluding those rated as at a higher risk of bias. The potential publishing bias was estimated Egger regression test. For studies that were not the lowest category in the reference group, we reclassified the lowest category as a reference and recalculated the impact using the Orsini method (18). We took the midpoint of the upper and lower boundaries of each type of sleep duration as the average level. If studies with open boundaries, we multiplied or divided the reported boundary by 1.25. Stata V.15.1 (StataCorp LLC, USA) was used to conduct the dose-response analyses.

## Results

### Literature search

**FIGURE 1:**
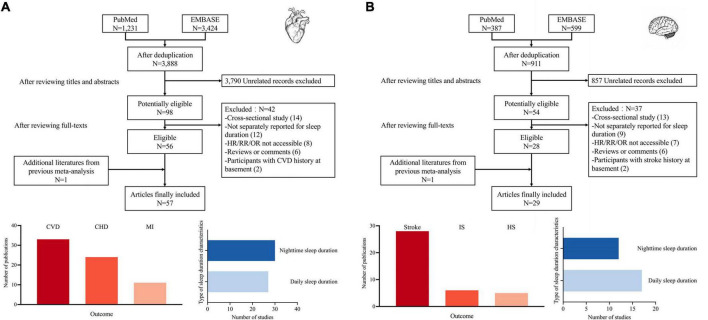
Search flowchart and summary characteristics of included studies, **(A)** for cardiovascular diseases and **(B)** for cerebrovascular diseases. CVD, cardiovascular disease; CHD, coronary heart disease; MI, myocardial infarction; IS, ischemic stroke; HS, hemorrhage stroke.

The process of literature screen and selection is shown in Figure 1. As for cardiovascular disease (Figure 1A), the search yielded 3,888 articles after deduplication, among which 98 articles were considered potentially eligible after scanning the titles and abstracts. After reviewing the full text, 42 articles were further excluded and one additional article was supplemented. Finally, 57 articles were included, including 33 studies for any cardiovascular disease (CVD), 24 for CHD, and 11 for myocardial infarction (MI). As for cerebrovascular disease (Figure 1B), the search yielded 911 articles after deduplication, among which 54 articles were considered potentially eligible. Finally, 29 articles were included, including 28 studies for stroke, 6 for ischemic stroke and 5 for hemorrhage stroke.

### Study characteristics

The detailed characteristics of cardiovascular disease studies are summarized in Table 1. All studies are cohort studies with populations from Asian Pacific (15 in China, 7 in Japan, 2 in South Korea, 1 in Singapore, and 1 in Australia), Europe (5 in Sweden, 4 in the UK, 2 in Finland, 1 in Denmark, 1 in Germany, 1 in Italy, 1 in the Netherlands, 1 in Russia and 1 in Europe), and North America (13 in US and 1 in Canada). The mean age ranges from 51 to 77 years old and the mean follow-up duration varied extensively from 0.75 to 34 years. Table 2 shows the detailed characteristics of cerebrovascular disease studies. All studies are cohort studies with populations from Asia (7 in China, 6 in Japan, and 2 in Singapore), Europe (2 in Sweden, 2 in Europe, 1 in Germany, and 1 in the UK), and North America (6 in the US). The mean age is varied from 45.7 to 64.5 years and the mean follow-up is varied from 0.75 to 16 years. The average study quality is moderate (median score = 6.5).

**TABLE 1:** Characteristics of 57 studies about cardiovascular diseases included in the meta-analysis.

*N*	References	Country	No of participants	Mean age	Female (*%)	Follow-up duration (years)	Outcome (No. of cases)	Sleep categories	NOS
1	Zhu et al. (19)	China	607	62.9	58.2	3.9 (mean)	CVD (105)	24-h sleep	6
2	Lian et al. (20)	China	873	61.6	30	1.25 (mean)	MI (314)	Nighttime sleep	5
3	Kario et al. (21)	Japan	2,236	63	49.6	7.1 (mean)	CAD (81)	Nighttime sleep	7
4	Tao et al. (4)	China	12,532	38–73	53.93	8.57 (mean)	CVD (16,541)	24-h sleep	6
5	Ye et al. (22)	China	8,968	56.7	65.3	0.75 (mean)	MI (102)	24-h sleep	6.5
6	Krittanawong et al. (23)	US	32,152	45.8	51.8	11 (mean)	CAD (242)	Nighttime sleep	7
7	Fan et al. (24)	UK	385,292	33–73	56.5	8.5 (mean)	CAD (4,667)	24-h sleep	7.5
8	Wang et al. (5)	China	52,599	52.5	23.8	6.7 (mean)	CVD (2,406)	Nighttime sleep	7
9	Li et al. (6)	China	2,687	61.2	19.1	3.7 (mean)	CVD (436)	24-h sleep	5
10	Kwon et al. (7)	Korea	34,264	≥ 20	57.1	6.3 (mean)	CVD (216)	24-h sleep	7.5
11	Daghlas et al. (25)	UK	461,347	40–69	46	7.04 (mean)	MI (5,218)	24-h sleep	6.5
12	Bochkarev et al. (26)	Russia	20,359	25–64	61.6	2 (mean)	CAD (2,116); MI (443)	24-h sleep	4.5
13	Xiao et al. (8)	US	55,375	40–79	61.1	14 (max)	CVD (2,631)	24-h sleep	6
14	Wang et al. (9)	China	116,632	35–70	58.2	7.8 (mean)	CVD (4,365)	Nighttime sleep	5.5
15	Kim et al. (10)	US	2,846	64 (mean)	38	2.8 (mean)	CVD (251)	Nighttime sleep	7
16	Lao et al. (28)	China	28,040	50.6	53.7	18 (mean)	CHD (2,740)	24-h sleep	6.5
17	Svensson et al. (27)	Sweden	16,344	45–73	57.4	16.5 (max)	CHD (1,748)	Nighttime sleep	7
18	Khan et al. (29)	Finland	1,734	42–61	0	25.9 (mean)	CHD events (202)	24-h sleep	6
19	Kobayashi et al. (11)	Japan	39,239	≥20	49.6	5 (mean)	CVD (365)	24-h sleep	5
20	Bertisch et al. (12)	US	4,437	64	53.5	11.6 (mean)	CVD (818)	Nighttime sleep	6.5
21	Strand et al. (32)	China	392,164	40.4	51.1	9.7 (mean)	CHD mortality (711)	Nighttime sleep	7
22	Gianfagna et al. (33)	Italy	2,722	35–74	0	17 (mean)	CHD (213); CVD (293)	Nighttime sleep	5.5
23	Wang et al. (31)	China	96,903	51.33	20.4	3.98 (mean)	MI (423)	Nighttime sleep	8
24	Yang (30)	China	19,370	62.8	55.9	4.2 (mean)	CAD (2,058)	Nighttime sleep	5.5
25	Cai et al. (34)	China	113,138	M:40–75; F:44–79	60.6	M:6.07 (mean); F:7.12 (mean)	CVD (1,389)	24-h sleep	7
26	Liu et al. (37)	Canada	3,086	≥30	52.3	20 (mean)	CHD (491)	24-h sleep	6
27	Xiao et al. (35)	US	239,896	51–72	43.8	14 (mean)	CVD (11,635)	Nighttime sleep	5.5
28	Rod et al. (36)	UK	9,098	35–55	32.8	22 (mean)	CVD (221)	Nighttime sleep	6
29	Canivet et al. (38)	Sweden	Male: 5,875 Female: 7,742	45–64	56.9	12 (mean)	CVD (1,602)	Nighttime sleep	6.5
30	Bellavia et al. (39)	Sweden	70,973	45–83	46.7	15 (mean)	CVD (3,981)	Nighttime sleep	7.5
31	Westerlund et al. (41)	Sweden	41,192	>18	64.5	13.2 (mean)	CVD (857); MI (1,908)	24-h sleep	7.5
32	Sands-Lincoln et al. (42)	US	86,329	50–79	100	10 (mean)	CVD (7257); CHD (5,359)	Nighttime sleep	7
33	Kakizaki et al. (45)	Japan	49,256	40–79	52.2	10.8 (mean)	CVD (2,549); IHD (561)	24-h sleep	8
34	Hale et al. (47)	US	3,942	50–79	100	16 (max)	CHD (132)	Nighttime sleep	6.5
35	Garde et al. (48)	Denmark	4,943	40–59	0	30 (mean)	IHD (587)	24-h sleep	7
36	Kim et al. (44)	US	135,685	45–75	54.4	12.9 (mean)	CVD (6,610); CHD (3,476); MI (1,188)	24-h sleep	7
37	Yeo et al. (40)	Korea	13,164	54.9	58.7	9.44 (mean)	CVD (363)	24-h sleep	6.5
38	Li et al. (43)	China	12,489	20–79	61.8	7 (mean)	CVD (312)	Nighttime sleep	6
39	Holliday et al. (46)	Australia	156,902	≥45	52.7	2.3 (mean)	CVD (4,852)	Nighttime sleep	7
40	Chen et al. (49)	China	4,064	73.8	44.2	7 (mean)	CVD (259)	Nighttime sleep	7
41	von Ruesten et al. (50)	Europe	23,620	35–65	61.4	7.8 (mean)	MI (197)	24-h sleep	7.5
42	Hoevenaar-Blom et al. (52)	Netherland	20,432	20–65	54.9	11.9 (mean)	CHD (1,148); CVD (1,486)	24-h sleep	7
43	Kronholm et al. (51)	Finland	22,484	44.5	51.2	29–34	CVD (3,174)	Nighttime sleep	5
44	Hamazaki et al. (53)	Japan	2,282	35–54	0	14 (mean)	CVD (64)	24-h sleep	5.5
45	Chandola et al. (55)	England	8,998	35–55	33.1	15 (mean)	CHD (1,205)	Nighttime sleep	7
46	Amagai et al. (56)	Japan	11,367	18–90	61.2	10.7 (mean)	CVD (481); MI (80)	Nighttime sleep	6.5
47	Chien et al. (54)	China	3,430	≥35	52.7	15.9 (mean)	CVD (420)	Nighttime sleep	6
48	Ikehara et al. (59)	Japan	98,634	40–80	57.9	14.3 (mean)	CVD (4,287); CHD (881)	24-h sleep	6.5
49	Suzuki et al. (57)	Japan	11,395	65–85	50.4	5.3 (mean)	CVD (310)	Nighttime sleep	6
50	Stone et al. (58)	US	8,101	≥69	100	7 (mean)	CVD (723)	Nighttime sleep	7
51	Shankar et al. (60)	Singapore	58,044	45–74	55.9	13 (mean)	CHD mortality (1,416)	24-h sleep	7
52	Meisinger et al. (61)	Germany	6,896	45–74	49.1	10.1 (mean)	MI (380)	Nighttime sleep	7
53	Lan et al. (62)	China	3,079	≤64	49.7	8.4 (mean)	CVD (379)	Nighttime sleep	6.5
54	Patel et al. (63)	US	82,969	30–55	100	14 (mean)	CVD (1,084)	24-h sleep	7.5
55	Ayas et al. (64)	US	71,617	40–65	100	10 (mean)	CHD (934)	24-h sleep	6.5
56	Mallon et al. (65)	Sweden	1,870	45–65	51.6	12 (mean)	CAD mortality (91)	Nighttime sleep	4.5
57	Qureshi et al. (66)	US	7,844	>31	63.7	10 (mean)	CHD (413)	Nighttime sleep	6

**TABLE 2:** Characteristics of 29 studies about cerebrovascular diseases included in the meta-analysis.

*N*	First author	Country	No of participants	Mean age	Female (*%)	Follow-up duration (years)	Outcome (No. of cases)	Sleep categories	NOS
1	Kario et al. (21)	Japan	2,236	63	49.6	7.1 (mean)	Stroke: (52)	Nighttime sleep	6
2	Zhao et al. (67)	China	4,204	63.8	52.9	11 (mean)	Ischemic Stroke (129)	Nighttime sleep	6.5
3	Ye et al. (22)	China	8,968	56.7	65.3	0.75 (mean)	Stroke (250)	24-h sleep	7
4	Krittanawong et al. (23)	US	32,152	45.8	51.8	11 (mean)	Stroke (1,157)	Nighttime sleep	7
5	Fan et al. (24)	UK	385,292	33–73	56.5	8.5 (mean)	Stroke (2,650)	24-h sleep	7.5
6	Titova et al. (69)	Sweden	79,881	45–79	44.7	14.6 (mean)	Stroke (8,091)	24-h sleep	6.5
7	Zhou et al. (68)	China	31,750	61.7	55.9	6.2 (mean)	Stroke (1,557)	Nighttime sleep	6
8	Nutakor et al. (70)	Ghana	3,617	≥50	46.47	3 (mean)	Stroke (82)	Nighttime sleep	7
9	Li et al. (6)	China	2,687	61.2	19.1	3.7 (mean)	Stroke (135)	24-h sleep	7
10	Ji et al. (71)	China	27,712	45.7	52.2	7 (mean)	Stroke (617)	Nighttime sleep	7.5
11	Bochkarev et al. (26)	Russia	20,359	25–64	61.6	2 (mean)	Stroke (422)	24-h sleep	5.5
12	Petrov et al. (72)	US	16,733	≥45	57.8	6.1 (mean)	Stroke (460)	Nighttime sleep	6
13	Kawachi et al. (75)	Japan	27,896	≥35	54	16 (mean)	Stroke (611)	24-h sleep	6
14	Song et al. (73)	China	95,023	18–98	20.6	7.9 (mean)	Stroke (3,135)	Nighttime sleep	6.5
15	Smagula et al. (74)	Singapore	8,265	64.59	58.95	12.7 (mean)	Stroke (535)	24-h sleep	7.5
16	Cai et al. (34)	China	113,138	40–74	54.9	7.12 (max)	Stroke (746)	24-h sleep	7
17	Helbig et al. (77)	Germany	12,131	25–74	49.2	14 (mean)	Stroke (826)	24-h sleep	7
18	Leng et al. (76)	Europe	9,692	42–81	54.1	9.5 (mean)	Stroke (346)	24-h sleep	6
19	Pan et al. (79)	Singapore	63,257	45–74	55.8	14.7 (mean)	Stroke (1,381)	24-h sleep	7
20	Ruiter Petrov et al. (78)	US	5,666	≥45	55.9	3 (mean)	Stroke (224)	Nighttime sleep	5.5
21	Westerlund et al. (41)	Sweden	41,192	>18	64.5	13.2 (mean)	Stroke (1,685)	24-h sleep	6
22	Kakizaki et al. (45)	Japan	49,256	40–79	52.2	10.8 (mean)	Stroke (1,165)	24-h sleep	7
23	Kim et al. (44)	US	135,685	45–75	54.4	12.9 (mean)	Stroke (1,259)	24-h sleep	6.5
24	von Ruesten et al. (50)	Europe	23,620	35–65	61.4	7.8 (mean)	Stroke (169)	24-h sleep	7
25	Hamazaki et al. (53)	Japan	2,282	35–54	0	14 (mean)	Stroke (30)	24-h sleep	7
26	Amagai et al. (56)	Japan	11,367	18–90	61.2	10.7 (mean)	Stroke (411)	Nighttime sleep	6.5
27	Ikehara et al. (59)	Japan	98,634	40–80	57.9	14.3 (mean)	Stroke (2,964)	24-h sleep	6
28	Chen et al. (80)	US	93,175	50–79	100	7.5 (mean)	Stroke (1,166)	Nighttime sleep	5.5
29	Qureshi et al. (66)	US	7,844	>31	63.7	10 (mean)	Stroke (285)	Nighttime sleep	6

### Sleep duration and risk of cardiovascular disease

**FIGURE 2:**
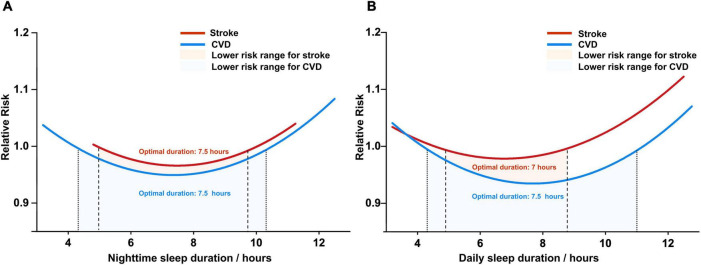
The dose-response analysis of cardio-cerebrovascular disease and sleep duration, **(A)** for nighttime sleep duration and **(B)** for daily sleep duration. CVD, cardiovascular disease.

Overall, 57 articles with 3.2 million participants about CVD were included in our meta-analysis (4–12, 19–66). A U-shaped relationship was found between CVD and the nighttime sleep duration category (*P*_*non–linearity*_ < 0.0001; Figure 2A). Specifically, we found the nighttime sleep duration associated with lower risk was roughly situated between 4.3 and 10.3 h/night (Figure 2A) with the lowest risk corresponding to roughly 7.5 h/night. As for daily sleep duration, findings were similar to what was described above: the optimal sleep duration was roughly 7.5 h per day. (*p*_*non–linearity*_ < 0.0001; Figure 2B).

In total, 24 articles were included in our study of CHD and sleep duration, and 11 articles were included in MI. A U-shaped relationship was found between CHD and nighttime sleep duration (*p*_*non–linearity*_ = 0.0001; Supplementary Figure 2), with a lower risk of CHD at 4.5–10.8 h which the optimal duration at approximately 7 h/night. The non-linear relationship between nighttime duration and MI showed non-significant significance (*p* = 0.2268), probably due to the limited number of included studies. Similarly, the dose-response analyses revealed significant non-linear associations between sleep duration and risks of CHD (*p* < 0.0001 for the daily duration) or MI (*p* = 0.0001 for the daily duration). Specifically, as for the daily sleep duration, the optimal duration was found to be roughly 7.5 h for the lowest risk of CHD and 7 h for lower risk of MI (Supplementary Figure 2). The risk of CHD or MI would be significantly elevated when the daily sleep duration was over 11 h or less than 4 h (4.6–11 for CHD and 4–10.6 for MI). Subgroup analysis showed that the results were consistent with the above results after stratification by region (*p*_*non–linearity*_ < 0.0001 for Asian Pacific, Europe and North America), follow-up duration (*p*_*non–linearity*_ < 0.0001 for < 10 and ≥ 10 years), and gender (*p*_*non–linearity*_ < 0.0001 for male and female) (Supplementary Figures 4–6).

### Sleep duration and risk of cerebrovascular disease

A total of twenty-nine studies related to cerebrovascular disease with 1.4 million participants were included in our study (6, 21–24, 26, 34, 41, 44, 45, 50, 53, 56, 59, 66–80). The dose-response analyses revealed significant U-shaped association between sleep duration and risk of cerebrovascular disease (*p*_*non–linearity*_ = 0.05 for nighttime and *p*_*non–linearity*_ < 0.0001 for the daily duration). Specifically, as for the nighttime sleep duration, the optimal duration was found to be roughly 7.5 h for the lowest risk of cerebrovascular disease (Figure 2A). The risk of cerebrovascular disease will be significantly elevated when the nighttime sleep duration is over 9.7 h or less than 5 h. Similarly, as for daily sleep duration, our study noted that the optimal sleep duration of cerebrovascular disease was roughly 7 h/day. Subgroup analysis showed that the results were consistent with the above results after stratification by region (*p*_*non–linearity*_ < 0.0001 for Asian Pacific, *p*_*non–linearity*_ = 0.0052 for Europe, and *p*_*non–linearity*_ = 0.0002 for North America), follow-up duration (*p*_*non–linearity*_ = 0.0077 for < 10 years and *p*_*non–linearity*_ < 0.0001 for ≥ 10 years) (Supplementary Figures 4, 5).

### Sensitivity analysis and publication bias

Sensitivity analysis excluded low-quality studies in turn did not alter the combined RR. Egger regression tests for evidence of publication bias were discussed extensively, (*p* = 0.010 for short sleep duration and total cardiovascular disease; *p* = 0.040 for short sleep duration and stroke) and we found that potential publication bias existed, but there was no change in the pooled results after correction.

## Discussion

Based on our understanding, this paper is the most extensive and largest study of the correlation between sleep duration and cardiovascular events (Supplementary Table 4). The optimal sleep duration (hours/night) was found to be linked to the lowest risk of cardiovascular disease. Additionally, its reminder feature evaluates oversleeping or sleep deprivation that could increase one’s risk. It is worth noting that estimating the relative risk only represents the study population (not in the analysis). Thus, it may significantly impact an individual.

As observed above, sleep duration was also linked to the increased risk of diseases beyond CCDs. From the standpoint of one’s overall health, it is vital to conduct further research on the protective nature of an optimal sleep duration range (4.7–8.9 h/day) in addressing CCDs against other diseases. Upon searching the keywords “dose-response,” “meta-analysis,” and “sleep duration” on the PubMed database, seven papers were found to delve into a dose-response connection with other diseases, such as metabolic syndrome, cognitive disorder, colorectal cancer, breast cancer, osteoporosis, all cancer types, and AD. For comparative purposes, 5–9.7 h/night was identified to still shield a person from all diseases, which was aligned with our research (Supplementary Figure 1).

Compared with the previously circulated meta-analyses (81–83), the advantages of this study are fourfold. First, a total of 27 new included studies have been added to achieve a significantly expanded sample size. Second, instead of summarizing risk estimates based only on extreme classifications (lowest and highest) and their comparisons, which could cause significant differences in the findings, dose-response meta-analysis should be the primary choice when performing a systematic review. Third, the two sleep categories were the daily and nighttime sleep durations. We analyzed the dose-response relationship between them and CCDs independently. Fourth, as detailed in Supplementary Table 1, more robust and enhanced NOS evaluation criteria were adopted.

In light of the sleep duration and adverse outcomes, a few mechanisms could influence their direct relationship. Several studies have demonstrated that a chronic stressor like short sleep duration could impact the biological activities of adipokines (adiponectin, ghrelin, and leptin) and the hormonal regulators of appetite. Consequently, it increases the risk of obesity, which has been proven to be one of the risk factors for CCDs. Bain et al.’s research exhibited the link of short sleep duration to impaired NO-mediated endothelium-dependent vasodilation (84), which increases CCD-related risks. Moreover, a robust predictor of CCDs is the inflammatory marker CRP, which, along with other pro-inflammatory markers (IL-17, –6, –1, and TNF-α), have been observed to rise after being sleep-deprived (85, 86). Regarding insomnia with short sleep duration, patients experience a significant weakening of their parasympathetic nerve activation and an increase in their sympathetic nerve imbalance (87). Lastly, habitually short sleep duration results in circadian rhythm disruption, affecting physiological functions like the diurnal blood pressure variation (88), which increases the risk of hypertension. These risk factors are collectively relevant to CCDs.

Furthermore, the following factors may be associated with the link mechanism of long-term sleep with the increased risk of CCDs. Pulse wave velocity (PWV) and cerebral small vascular disease have a close relationship (89). Nevertheless, long sleep duration serves as a fundamental determinant of the incidence of stroke resulting from the PWV increase among those with a higher risk of CCDs (90). The increased risk of cardiometabolic dysfunction is associated with both long and short sleep durations (91). Over time, having a long sleep duration leads to lower levels of physical activity, causing poor health and physical weakness (92).

There are four primary limitations to our paper. First, the correlation deduced from analyzing observational cohort studies does not imply causation. To address this, future research must utilize Mendelian randomization techniques to evaluate the role of sleep duration management in CCD prevention. Second, compared with the gold standard of polysomnography that accurately measures sleep duration, the self-reported data across all the studies may not qualify as an objective measurement. However, due to its high costs, the former method may be unfeasible in large-scale prospective cohort studies. Meanwhile, an existing paper validated the moderate correlation between the sleep duration that was self-reported and measured by a wrist monitor (*r* = 0.47). Third, participants in most studies may have modified their sleep patterns after one follow-up, which assessed their respective sleep duration. As a result, the continuous effect of sleep duration on the long-term risk of CCD may not be fully captured by a single exposure measurement. Fourth, given the small sample size, we were unable to quantify the link of nap duration to CCDs through the dose-response analysis. Ultimately, recent literature has exhibited that sleep duration and sleep onset timing are related to CCDs (93), providing a new focus for our follow-up research.

In summary, our study provides varying degrees of evidence, indicating that the adults sleeping 5–9.7 h per night can reduce the risk of both CCDs, with the lowest risk with 7.5 h per night of sleep duration. Future research needs to confirm the relationship with cardio-cerebrovascular diseases and examine the role of sleep management in improving the risk of CCDs.

## Data availability statement

The original contributions presented in this study are included in the article/Supplementary material, further inquiries can be directed to the corresponding author/s.

## Author contributions

Y-MH: collection and analysis of the data, drafting and revision of the manuscript, and prepared all the figures. WXi: collection and analysis of the data and revision of the manuscript. Y-JG and J-HH: revision of the manuscript. WXu, C-CT, and LT: conceptualization and design of the study and revision of the manuscript. All authors contributed to the article and approved the submitted version.
