# Supplementary material for: Sleep duration and risk of cardio-cerebrovascular disease: A dose-response meta-analysis of cohort studies comprising 3.8 million participants
Source: Front Cardiovasc Med. 2022 Sep 27;9:907990. doi: 10.3389/fcvm.2022.907990 (PMC9551171; doi:10.3389/fcvm.2022.907990)
Supplement: Supplementary file 1 [file Data_Sheet_1.PDF]

Content

Search formula： 2

Figure S1 The optimal sleep duration for dose-response analysis of nine diseases 3

Figure S2 Non-linear dose-response analysis of daily sleep duration and CHD&MI by sleep duration category. 5

Figure S3. Non-linear dose-response analysis of sleep duration and total cardiovascular disease by sleep duration category. Nighttime sleep duration (A) and 24-hour sleep duration (B) for cardiovascular disease, Nighttime sleep duration (C) and 24-hour sleep duration (D) for cerebrovascular disease. 6

Figure S4. Non-linear dose-response analysis of sleep duration with total cardiovascular disease and cerebrovascular disease by continent. Asia-Pacific (A), Europe (B) and North America (C) for cardiovascular disease. Asia (D), Europe (E) and North America (F) for cerebrovascular disease. 7

Figure S5. Non-linear dose-response analysis of sleep duration with total cardiovascular disease and cerebrovascular disease. Follow-up duration <10 years (A) and follow-up duration ≥10 years (B) for cardiovascular disease, Follow-up duration <10 years (C) and follow-up duration ≥10 years (D) for cerebrovascular disease. 8

Figure S6. Non-linear dose-response analysis of sleep duration and total cardiovascular disease by male (A), female (B) 9

Table S1 Newcastle-Ottawa Quality Assessment Scale- Cohort Studies (involving version)[& 1,2] 10

Table S2 Sleep duration and cardiovascular disease 12

Table S3 Sleep duration and cerebrovascular disease 19

Table S4 Comparison between previous meta-analysis and the present study. Cardiovascular disease (A); Cerebrovascular disease (B). 23

Search formula：

(((((((((cohort) OR (longitudinal)) OR (prospective)) OR (retrospective)) OR (nested case-control))) AND ((((stroke) OR (cerebral infarction)) OR (cerebral hemorrhage))) OR (cerebrovascular accident)))) AND (sleep duration); ((((((((((((cohort) OR (longitudinal)) OR (prospective)) OR (retrospective)) OR (nested case-control))) AND (((((((CVD) OR (cardiovascular disease)) OR (coronary heart disease))) OR (myocardial infarction)))) OR(CHD))))) OR(MI))))))) AND (sleep duration).

Figure S1 The optimal sleep duration for dose-response analysis of nine diseases

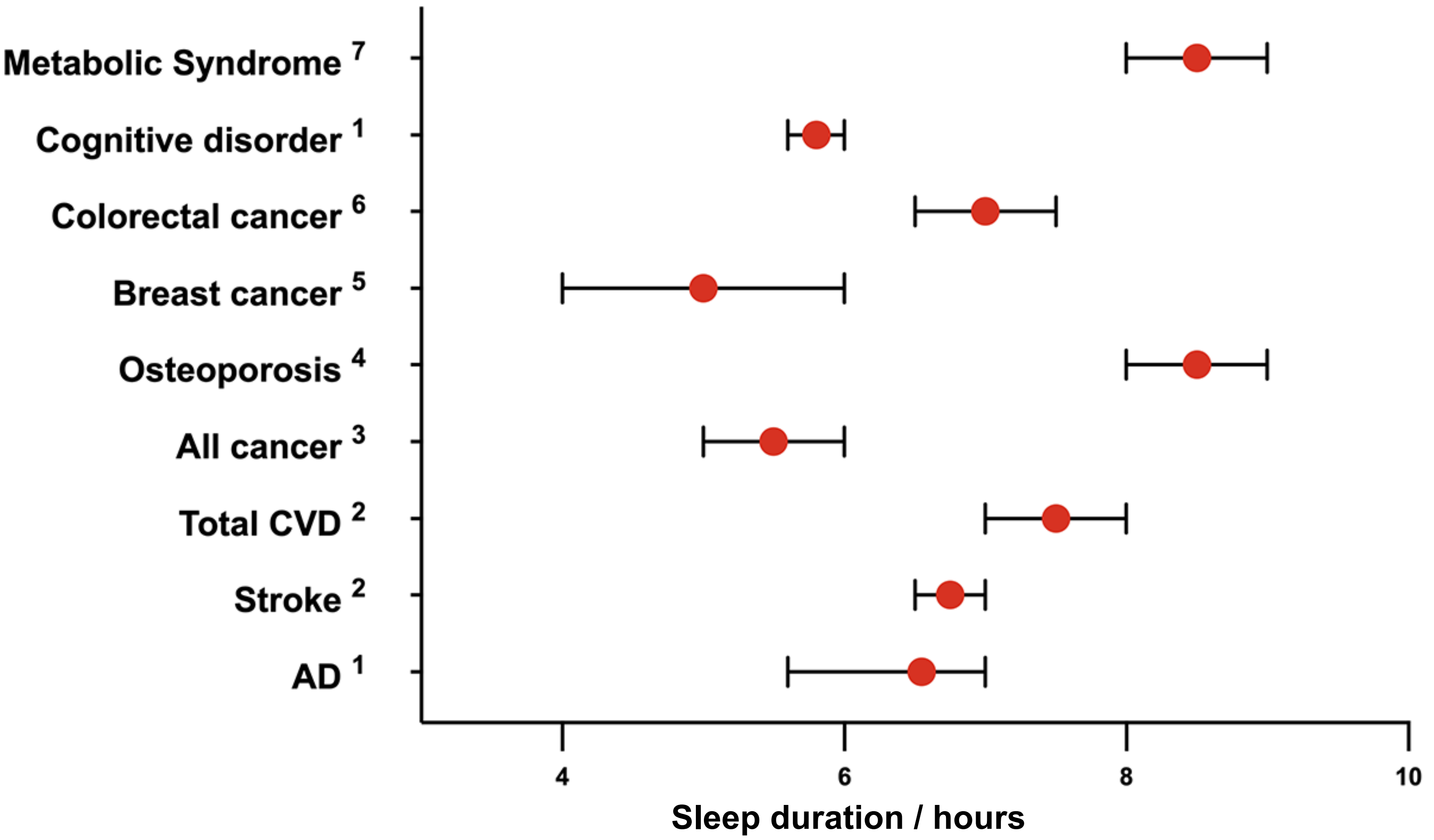

Reference

1. Chen Y, Tan F, Wei L, et al. Sleep duration and the risk of cancer: a systematic review and meta-analysis including dose-response relationship. *BMC Cancer*. Nov 21 2018;18(1):1149. doi:10.1186/s12885-018-5025-y
2. This study
3. Iftikhar IH, Donley MA, Mindel J, Pleister A, Soriano S, Magalang UJ. Sleep Duration and Metabolic Syndrome. An Updated Dose-Risk Metaanalysis. *Ann Am Thorac Soc*. Sep 2015;12(9):1364-72. doi:10.1513/AnnalsATS.201504-190OC
4. Li Y, Cai S, Ling Y, et al. Association between total sleep time and all cancer mortality: non-linear dose-response meta-analysis of cohort studies.

Sleep Med. Aug 2019;60:211-218. doi:10.1016/j.sleep.2019.03.026
5. Lu C, Sun H, Huang J, et al. Long-Term Sleep Duration as a Risk Factor for Breast Cancer: Evidence from a Systematic Review and Dose-Response Meta-Analysis. *Biomed Res Int*. 2017;2017:4845059. doi:10.1155/2017/4845059
6. Wang D, Ruan W, Peng Y, Li W. Sleep duration and the risk of osteoporosis among middle-aged and elderly adults: a dose-response meta-analysis. *Osteoporos Int*. Aug 2018;29(8):1689-1695. doi:10.1007/s00198-018-4487-8
7. Xu W, Tan CC, Zou JJ, Cao XP, Tan L. Sleep problems and risk of all-cause cognitive decline or dementia: an updated systematic review and meta-analysis. *J Neurol Neurosurg Psychiatry*. Mar 2020;91(3):236-244. doi:10.1136/jnnp-2019-321896

Figure S2 Non-linear dose-response analysis of sleep duration and CHD&MI by sleep duration category. CHD and nighttime sleep duration (A); CHD&MI with daily sleep duration (B).

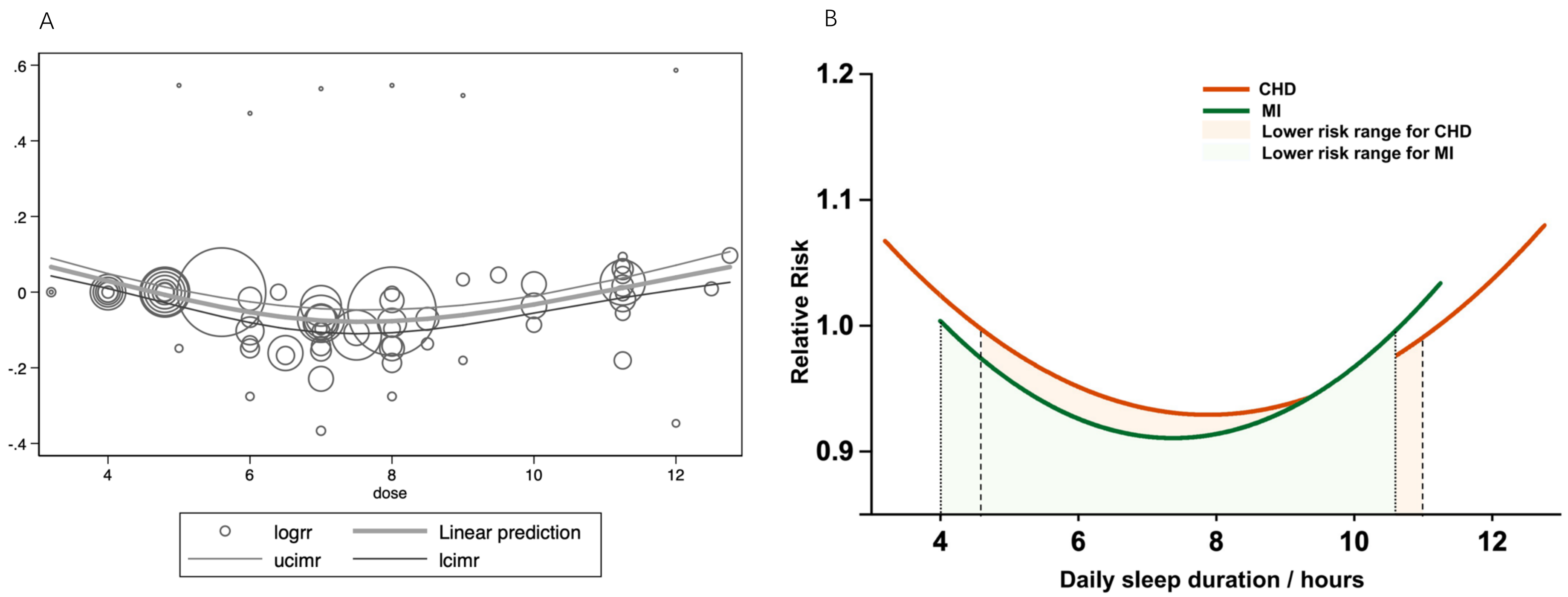

Figure S3. Non-linear dose-response analysis of sleep duration and total cardiovascular disease by sleep duration category. Nighttime sleep duration (A) and 24-hour sleep duration (B) for cardiovascular disease, Nighttime sleep duration (C) and 24-hour sleep duration (D) for cerebrovascular disease.

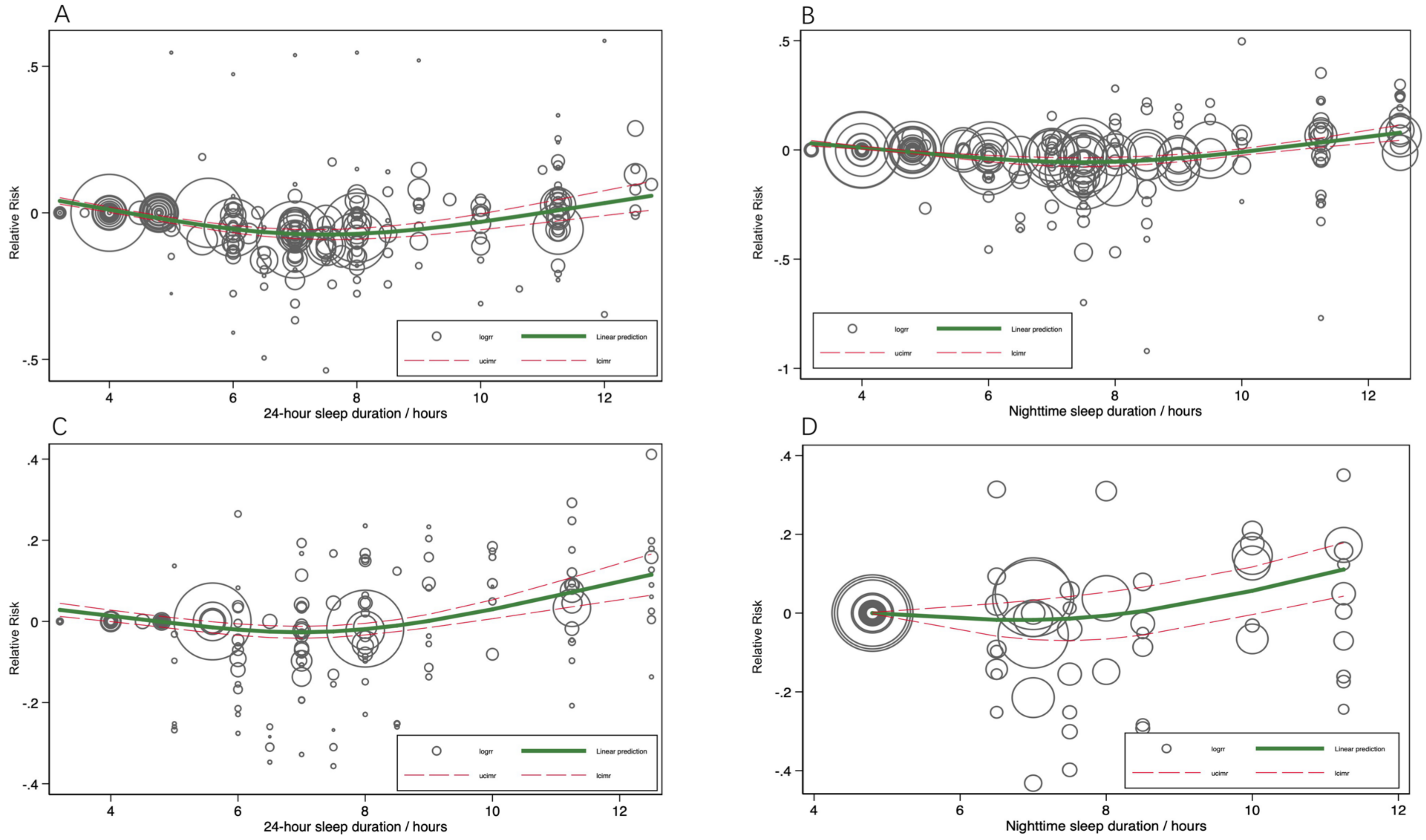

Figure S4. Non-linear dose-response analysis of sleep duration with total cardiovascular disease and cerebrovascular disease by continent. Asia-Pacific (A), Europe (B) and North America (C) for cardiovascular disease. Asia (D), Europe (E) and North America (F) for cerebrovascular disease.

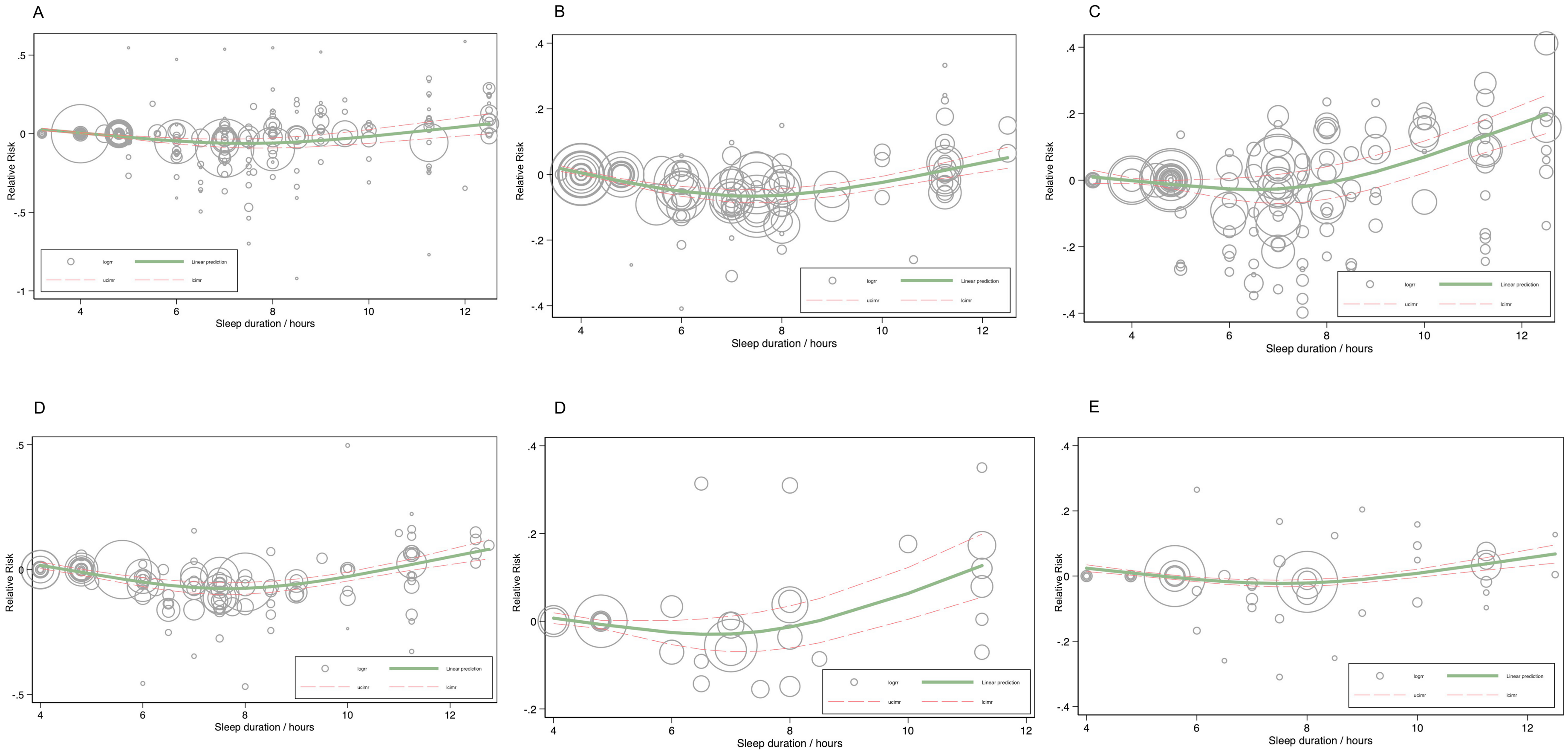

Figure S5. Non-linear dose-response analysis of sleep duration with total cardiovascular disease and cerebrovascular disease. Follow-up duration <10 years (A) and follow-up duration ≥10 years (B) for cardiovascular disease, Follow-up duration <10 years (C) and follow-up duration ≥10 years (D) for cerebrovascular disease.

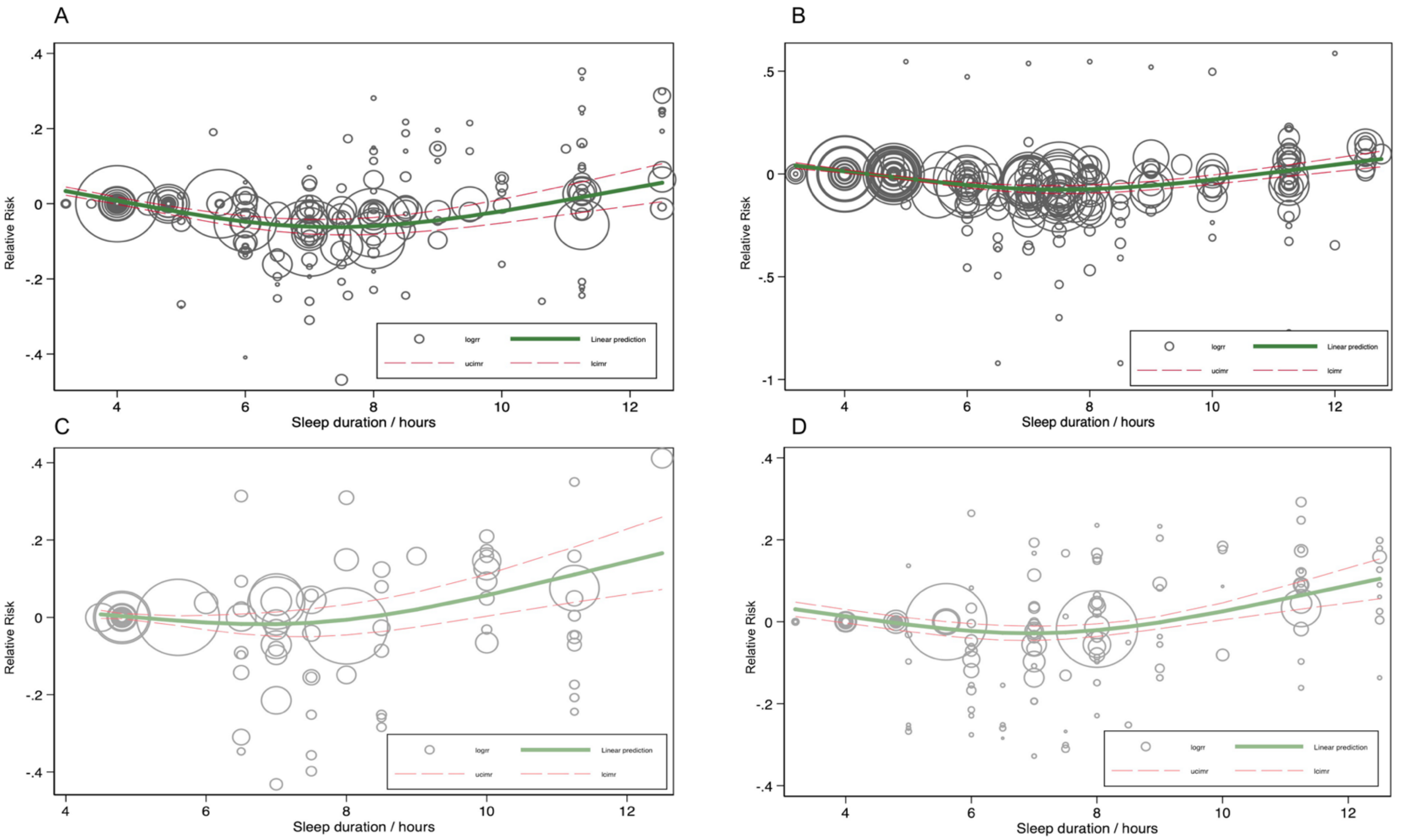

Figure S6. Non-linear dose-response analysis of sleep duration and total cardiovascular disease by male (A), female (B)

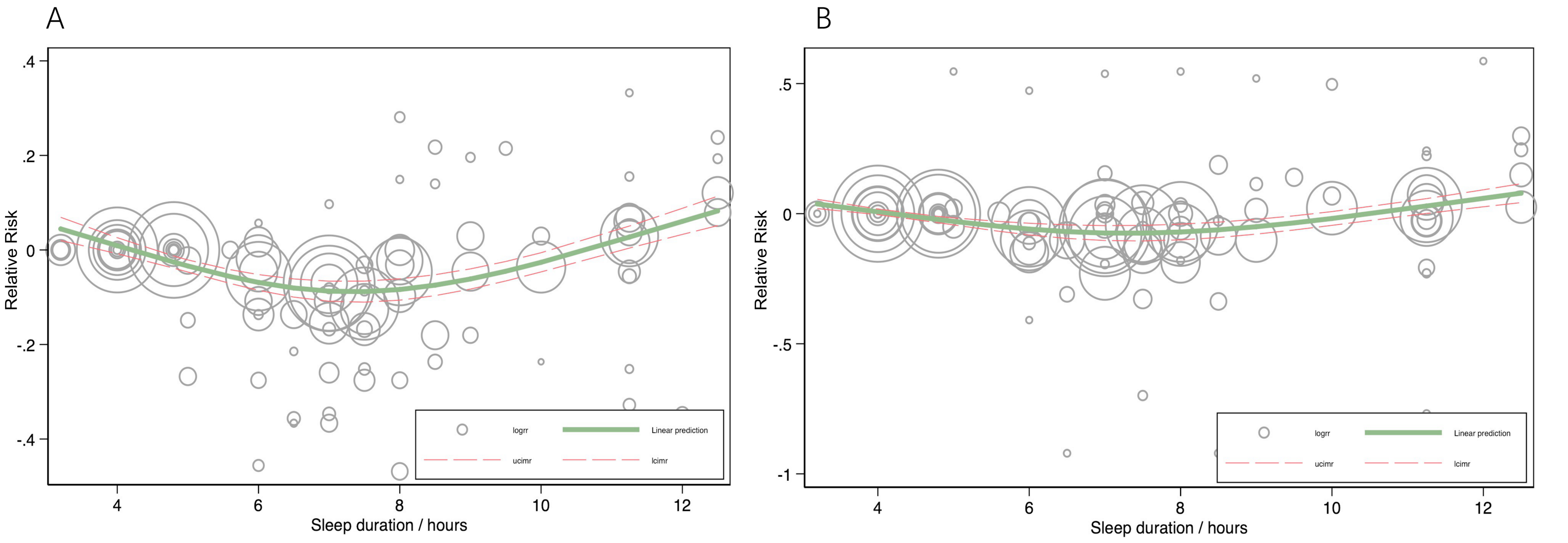

Table S1 Newcastle-Ottawa Quality Assessment Scale- Cohort Studies (involving version)[&] (Xu et al., 2020, Wells et al., 2000)

Risk of bias	Questions	Scores	Standards
**Selection (generalisability, assessment bias and potential reverse causality)**	Q1. representativeness of the exposed cohort	☆	a) randomly selected or<br>b) database covering very large population or<br>c) participation rate (PR) is ≥ 90% or<br>d) reported there is no difference in important characteristics between those who agreed to participate and those who did not
		0.5☆	PR varies from 70% to 90% with no reporting of significant difference in important characteristics between those who agreed to participate and those who did not
		0	Selected group of users e.g. nurses, volunteers or no description
	Q2. selection of the non-exposed cohort	☆	Drawn from the same community as the exposed cohort
		0.5☆	Self-report to simple question with potential recall bias
		0	Drawn from a different source or no description of the derivation of the non-exposed cohort
	Q3. ascertainment of exposure	☆	Questionnaire or interview based on self-report to series questions or database
		0.5☆	Self-report to simple question with potential recall bias
		0	No description
	Q4. demonstration that outcome of interest was not present at baseline	☆	Demonstration that outcome of interest was not present at start of study (cardio-cerebrovascular disease)
		0.5☆	Free of cardio-cerebrovascular disease (CVD & CHD & MI & stroke) for outcome of cardio-cerebrovascular disease
		0	No description
**Confounding bias**	Q5. comparability of cohorts on the basis of the design or analysis	☆☆	Except for age, sex, and education, the analysis still controls forat least another two domains of cardio-cerebrovascular disease risk factors, including smoking, BMI, physical activity, alcohol intake, depression, diabetes, high blood pressure, hyperlipidemia, comorbid conditions etc.
		☆	Controls for age, sex and education
		0	No description
**Outcome**	Q6. assessment of outcome	☆	Independent or blind assessment

**(assessment bias and attrition bias)**		0.5☆	Record linkage (e.g. identified through ICD codes on database records or claim data)
		0	Self-report or no description
	Q7. follow-up long enough for outcomes to occur?[#]	☆	The average or max duration ≥5 years.
		0	The average or max duration <5 years.
	Q8. adequacy of follow up of cohorts	☆	Attrition rate ≤ 5%
		0.5☆	5% ≤ Attrition rate ≤ 20%
		0	Attrition rate > 20% and no description of those lost or no description

& A study can be awarded a maximum of one star for each numbered item within the Selection and Outcome categories. A maximum of two stars can be given for Comparability. In the involving version, an assignment of a half point (0.5) is permitted.

Reference

Stang A. Critical evaluation of the Newcastle-Ottawa scale for the assessment of the quality of nonrandomized studies in meta-analyses.Eur J Epidemiol. 2010 Sep;25(9):603-5.

Xu W, Tan CC, Zou JJ, Cao XP, Tan L. Sleep problems and risk of all-cause cognitive decline or dementia: an updated systematic review and meta-analysis. *J Neurol Neurosurg Psychiatry*. Mar 2020;91(3):236-244. doi:10.1136/jnnp-2019-321896

Table S2 Sleep duration and cardiovascular disease

Author	Year	Sleep duration (h/d) and relative risk (95% CI)	Covariates in fully adjusted model	Endpoints (No. of cases)
Tao et al	2021	≤5, 1.23(1.16, 1.31); 6, 1.09(1.04, 1.13); 7, 1.0(reference); 8, 0.99 (0.95, 1.03); ≥9, 1.08(1.02, 1.15)	Age, gender, ethnicity, employment status, education level, TDI, physical activity, BMI, alcohol intake frequency, smoking status, history of hypertension and diabetes.	CVD (16,541)
Lian et al	2021	<6, 2.968 (1.951, 4.516); 6-9,1.0(reference); >9, 1.696 (0.795, 3.616)	Age, sex, BMI, residence, education level, history of diabetes, hypertension and dyslipidemia, history of coronary artery disease, use of statins, antiplatelet agents, β-blockers, ACEI or ARB, smoking, drinking, diet and regular exercise; female Adjusted for age, sex, BMI, residence, education level, history of diabetes, hypertension and dyslipidemia, history of coronary artery disease, use of statins, antiplatelet agents, β-blockers, ACEI or ARB, smoking, drinking, diet and regular exercise, and adjusted mutually for other sleep factors without significant relationships with each other	MI (314)
Kario et al	2021	<6, 1.61 (0.76,3.14); 6-9, 1.0(reference); ≥9, 0.97(0.46,2.05)	Age, sex, body mass index, current smoking, history of diabetes, statin use, aspirin use, antihypertensive medication use, history of cardiovascular disease, total cholesterol, high-density lipo- protein cholesterol, and office systolic blood pressure	CAD (81)
Wang et al	2020	<6, 1.04 (0.89-1.21) ; 6-7, 0.98 (0.88-1.08) ; 7-8, 1.0(reference); 8-9, 1.02 (0.92-1.14); ≥9, 1.10 (0.59-2.06)	Age, sex, maritalstatus, occupation, meanincome, educational attainment, physical activity, smoking status, alcohol consumption status, salt intake, family history of stroke, family history of myocardial infarction, hypertension, hyperlipidemia, diabetes, snoring frequency, sleep duration in 2010, antihypertensive use, hypoglycemic use, use of agents to lower lipid levels, body mass index, fasting blood glucose level, high-sensitivity C-reactive protein, systolic blood pressure, diastolic blood pressure, and estimated glomerular filtration rate.	CVD (2,406)
Li et al	2020	<6, 0.88 (0.64–1.23)； 6-8, 1.0(reference); ≥8, 0.98 (0.75–1.28)	Age, sex, continuous positive airway pressure adherence, disease type, baseline snore frequency, Epworth sleepiness scale score, Hospital Anxiety Depression Scale depression subscale score, and apnea–hypopnea index.	CVD (436)
Kwon et al	2020	≤4, 0.67 (0.38–1.20); 5-6, 1.04 (0.71–1.53); 7-8, 1.0(reference); ≥9, 1.20 (0.69–2.10)	Age, sex, marital status, education, occupation, household income, region, smoking status, physical activity, body mass index, alcohol drinking self-rated health and metabolic syndrome.	CVD (216)
Ye et al	2020	Without MetS: <6, 1.135 (0.342, 3.762); 6-7, 1.009 (0.485, 2.098); 7-8, 1.0(reference); 8-9, 1.067 (0.625, 1.820); >9, 0.675 (0.264, 1.727); With MetS: <6, 1.213 (0.358, 4.104); 6-7, 0.745 (0.286, 1.942); 7-8, 1.0(reference); 8-9, 1.677 (0.984, 2.855); >9, 1.731 (0.896, 3.344)	Age, sex, BMI, SBP, depression, blood glucose, triglycerides and physical activity	MI (102 with MetS:57; without MetS:45)

Krittanawong et al	2020	<7, 1.17 (0.99−1.39); 7-9, 1.0(reference); >9, 1.17 (0.86−1.56)	Age, gender, BMI, marital status, educational level, physical activity, sedentary activity, current cigarette smoking, depression, blood pressure, lipid profiles, and hemoglobin.	CAD (242)
Fan et al	2020	<7, 1.12 (1.05,1.20); 7-9, 1.0(reference); >9, 1.19 (1.08,1.32)	Age, sex, the Townsend Deprivation Index, ethnic, total physical activity level, smoking status, alcohol consumption, and family history of heart diseases or stroke.	CHD (4,667)
Xiao et al	2019	White race: <6, 1.32 (1.06, 1.65); 6, 1.11(0.91, 1.36); 7.5, 1.0(reference); 11.25, 1.15 (0.88, 1.52) Black race: <6, 1.07 (0.94, 1.22); 6, 1.05 (0.94, 1.18); 7.5, 1.0(reference); 11.25, 1.03 (0.90, 1.17)	Ag, sex, education, household income, marital status, and employment status, smoking, alcohol, total physical activity, total sitting time, BMI, and history of diabetes, hypertension, hypercholesterolemia and depression.	CVD (2,631)
Wang et al	2019	<6, 1.1(0.98,1.25); 6-8, 1.0(reference); 8-9, 1.04(0.96,1.12); 9-10, 1.1(1,1.21); >10, 1.28(1.14,1.44)	Age, sex, education attainment, smoking status, drinking status, urban or rural residency, family history of cardiovascular diseases (coronary heart disease and stroke), the history of diabetes, hypertension and chronic obstructive pulmonary disease, de- pression, and centre as random effect	CVD (4,365)
Kim et al	2019	<4.5, 2.04(1.19,3.49); 4.5-5.4, 1.08(0.62-1.87); 5.5-6.4, 1.53(1.02-2.31); 6.5-7.4, 1.0(reference); 7.5-8.4, 1.21(0.81-1.79); ≥8.5, 1.13(0.64-1.99)	Age, sex, race, body mass index, hypertension, diabetes, hyperlipidemia, smoking history, prior myocardial infarction, heart failure, acute myocardial infarction at enrollment, revascularization at enrollment, and obstructive coronary artery disease	CVD (251)
Daghlas et al	2019	4, 1.34(1.07-1.68); 5, 1.19 (1.06-1.35); 6, 1.05(0.98-1.13); 7-8, 1.0(reference); 9, 1.07 (0.96-1.19); 10,1.32(1.11-1.58); 11, 1.87(1.14-3.06)	Age, sex, ethnicity, smoking status, frequency of alcohol consumption, history of heart disease in family, marital status, education, income, Townsend deprivation index, employment status, physical activity (MET/h-week), television watching, grip strength, BMI, waist-hip ratio (WHR), history of seeing provider for mental health, snoring, use of sleep medications, self-reported or medical record derived sleep apnea, and self-reported insomnia, probable type 2 diabetes, hypertension, use of blood pressure lowering medication, history of high cholesterol, use of cholesterol lowering medication, and use of aspirin	MI (5,218)
Bochkarev et al	2019	CAD: ≤6, 1.3(1.2; 1.5); 6-7,0.9 (0.8; 1.0); 7-8, 1.0(reference); 8-9, 1.1 (0.9; 1.4); >9, 1.5 (1.1; 1.8) MI:≤6, 1.1 (0.9; 1.5); 6-7,0.8 (0.6; 1.0); 7-8, 1.0(reference); 8-9, 1.3 (0.9; 2.0); >9, 1.6 (1.02; 2.4)	Sex, age, body mass index, office blood pressure, smoking and low physical activity	CAD (2,116); MI (443)
Kobayashi et al	2018	<6, 1.76(1.02,3.04); 6-7, 1.12(0.7,1.11); 7-8, 1.0(reference); >8, 1.21(0.67,2.2)	Age, gender, occupation, health habits, marital status, calorie intake, treatment status outcomes and time variables	CVD (365)
Bertisch et al	2018	<6, 0.96(0.81,1.14); ≥6, 1.0(reference)	Propensity score adjusted, Hypertension, diabetes and lipids	CVD (818)
Lao at al	2018	M: <6,1.09 (0.96-1.25);6-8,1.00 (reference);>8, 1.14 (0.95-1.37); F: <6, 1.17 (1.05-1.32);6-8,1.00 (reference);>8, 1.08 (0.90-1.30)	Age, sex, educational level, marital status, alcohol drinking, cigarette smoking, vegetable intake, fruit intake, physical activity in leisure time, physical activity in work, and family history of cardiovascular disease.	CHD (M: 1,288; F:1,452)

Svensson at al	2018	M: <6, 1.41 (1.06, 1.89); 6-7, 1.12 (0.94, 1.33); 7-8, 1.0(reference); 8-9, 1.15 (0.99, 1.34); ≥9, 1.33 (1.01, 1.75) F: <6, 1.46 (1.03, 2.07); 6-7, 1.06 (0.83, 1.34); 7-8, 1.0(reference); 8-9, 0.96 (0.78, 1.19); ≥9, 1.32 (0.91, 1.91)	Age, socioeconomic index, marital status, smoking, alcohol intake, physical activity, hypertension, use of lipid-lowering medication, shift work, psychological stress, sleep quality and excluded the first 3 years of follow-up	CHD (1,748)
Khan at al	2018	<8 1.0(reference); 8.1-9 0.73(0.45-1.21); 9.1-10.2 1.11(0.77-1.60); >10.2 1.25(0.86-1.81)	Age, diabetes, smoking and alcohol use, body mass index, systolic blood pressure,serum creatinine and serum LDL-c, physical activity, serum C-reactive protein	CHD events (202)
Gianfagna et al	2016	≤6, 1.14(0.84,1.53); 7-8, 1(reference); ≥9, 1.55(1.08,2.21)	Age, systolic BP, total cholesterol, HDL cholesterol, diabetes, smoking habits, and educational level, sleep disturbances, LTPA and depression	CVD (293)
Strand at al	2016	M: <4,1.00(0.51-1.94);4-6,1.05(0.84-1.31);6-8,1.00 (reference);>8,1.17(0.92-1.48); F: <4,1.82(1.02-3.24);4-6,0.99(0.71-1.37);6-8,1.00 (reference);>8,1.95(1.38-2.76)	Age, sex, education and marital status, smoking, alcohol consumption, physical activity, history of hypertension, history of diabetes and history of heart diseases.	CHD mortality (M:489; F:222)
Gianfagna et al	2016	≤6 1.14(0.80–1.61); 7-8,1.0; ≥9 1.32(0.85–2.07)	Systolic BP, total cholesterol, HDL cholesterol, diabetes, smoking habits, and educational level, plus mutual adjustment for sleep disturbances and sleep duration LTPA and depression	CHD (213)
Wang at al	2016	≤5 0.89(0.60–1.30)； 6 0.84(0.61–1.16)； 7 1.0(reference); 8 0.86(0.66–1.13); ≥9 1.12(0.58–2.16)	Age, sex, family per member monthly income, education level, marital status, smoking status, drinking status, physical activity, history of hypertension, diabetes mellitus, and hyperlipidemia.	MI (423)
Li et al	2016	≤5, 1.43(0.87-2.33); 6-8,1.0(reference); ≥9, 1.70 (1.05-2.77)	All the potential risk factors for CHD (full model)	CHD (M: 203; F: 141)
Yang et al	2016	≤7, 1.08 (0.90-1.29); 7-<8,1.0 (reference); 8-<9, 1.04 (0.93-1.16); 9-<10, 1.03 (0.90-1.18); ≥10, 1.33 (1.10-1.62)	Age, sex, BMI, education, smoking status, drinking status, physical activity, hypertension, hyperlipidemia, diabetes, family history of CHD, and midday napping	CAD (2,058)
Cai et al	2015	4-5, 1.05(0.87,1.26); 6, 1.1(0.94,1.29); 7,1.0(reference); 8, 1.22(1.05,1.43); 9, 1.47(1.17,1.85); ≥10, 2.04(1.65,2.53)	Age, education, income, smoking, alcohol consumption, tea consumption, comorbidity score, history of night-shift work, participation in regular exercise, body mass index, and waist-to-hip ratio, cardiovascular disease, upper gastrointestinal tract	CVD (1,389)
Xiao et al	2014	<5,1.29(1.13,1.47); 5-6, 1.04(0.98,1.09); 7-8, 1.0(reference); ≥9,1.14(1.01,1.3)	Sex, age at baseline, race/ethnicity, marital status, education, self-reported health (excellent, very good, good, or fair), smoking, smoking dose; years since quitting smoking.	CVD (11,635)
Rod et al	2014	M: ≤6，1.18 (0.87-1.63);7-8,1.0(reference); >9, 1.61 (0.40-6.59); F: ≤6，1.81 (1.05-3.10);7-8,1.0(reference)	Age, employment grade, ethnicity, and marital status.	CVD (221)
Canivet et al	2014	M: ≤6,1.1 (0.96-1.3); 7-8,1.0(reference); ≥9,1.3 (1.01-1.7); F: ≤6, 1.3 (1.1-1.5); 7-8,1.0(reference); ≥9, 1.5 (1.1-2.1)	Age	CVD (1,602)
Bellavia et al	2014	<6, 1.44(1.20-1.73); 6-6.5, 1.23(1.09 to 1.38); 6.6–7.4, 1.0(reference); 7.5-8, 1.02(0.92-1.12); >8, 1.11(0.95 -1.31)	Sex, age at baseline, body mass index, smoking status and pack-years of smoking, alcohol consumption, and educational level.	CVD (3,981)

Liu et al	2014	≤6, 1.29 (1.03-1.61); 7-8, 1.0 (reference); ≥9, 1.13 (0.81-1.58)	Age, gender, current smoking, weekly alcohol drinking, SBP, total cholesterol levels, and BMI, diabetes, C-reactive protein	CHD (491)
Yeo et al	2013	≤5,1.4(1.02,1.23); 6,1.25(0.92,1.69); 7,1.0(reference); 8,1.04(0.76,1.42); 1.26, (0.81,1.96); 1.37, (0.82,2.29)	Age, sex, educational attainment, body mass index, cigarette smoking, alcohol consumption, past history of hypertension, type 2 diabetes, CVD, and metabolic syndrome.	CVD (363)
Westerlund et al	2013	≤5,1.11(0.76,1.64); 6, 1.17(0.88,1.55); 7,1.0(reference); ≥8, 1.12(0.85,1.47)	Age, sex, education, employment status, smoking, alcohol, snoring, work schedule, depressive symptoms, self-rated health, physical activity, BMI, diabetes, lipid disturbance, and hypertension	CVD (857)
Sands-Lincoln et al	2013	≤5，1.06(0.96,1.16); 6,1(0.95,1.06); 7-8, 1.0(reference); 9,0.95(0.83,1.08); ≥10, 1.23(0.89,1.7)	Age, race, education, income, smoking, BMI, physical activity, alcohol intake, depression, diabetes, high blood pressure, hyperlipidemia, comorbid conditions.	CVD (7,257)
Li et al	2013	M: ≤5, 1.57(0.35-7.15); 6, 0.6(0.17,2.15); 7,1.0(reference); 8,1.04(0.49,2.21); ≥9,2.73(1.22,6.11); F: ≤5, 0.80 (0.18-3.47); 6, 0.91 (0.38–2.23); 7,1.0(reference); 8,1.13 (0.57–2.23); ≥9, 1.72 (0.76–3.89)	Age, body mass index, systolic blood pressure, diastolic blood press, smoking status, drinking habits and physical activity.	CVD (312)
Kim et al	2013	M: ≤5，1.13 (1.00-1.28)； 6，1.01 (0.92-1.11)；7, 1.0(reference); 8, 1.05 (0.96-1.14); ≥9, 1.22 (1.09-1.35)<br>F: ≤5，1.20 (1.05-1.36)； 6，1.06 (0.96-1.18)；7, 1.0(reference); 8, 1.08 (0.98-1.20); ≥9, 1.29 (1.13-1.47)	Age as the time metric, adjusted for 5-year age groups at cohort entry, sex, ethnicity, education, marital status, history of hypertension or diabetes at enrollment, alcohol consumption, energy intake, body mass index, physical activity, hours spent daily watching television, and smoking history by inclusion of the following variables: [smoking status, average number of cigarettes, average number of cigarettes squared, number of years smoked (time dependent), number of years since quitting (time dependent), and interactions between ethnicity and the smoking variables].	Male: 3,772<br>Female: 2,838
Kakizaki et al	2013	≤6, 1.1(0.96,1.28); 7,1.0(reference); 8,1.21(1.08,1.36);9,1.32(1.15,1.52) ; ≥10, 1.49,(1.3,1.71)	Age; sex; total caloric intake; body mass index in kg m^2; marital status; level of education; job status; history of myocardial infarction; history of cancer; history of stroke; history of hypertension; history of diabetes mellitus; smoking status; alcohol drinking; time spent walking; perceived mental stress; self-rated health, physical function.	CVD (2,549)
Holliday et al	2013	<6, 1.03(0.88,1.21); 6, 1.06(0.96,1.17); 7, 1.0(reference); 8, 0.98(0.91,1.05); 9,0.98(0.89,1.09); ≥10,1(0.88,1.14)	Age, sex, education, marital status, residential remoteness, alcohol consumption, smoking status, health insurance status, income, body mass index, physical activity and baseline health status.	CVD (4,852)
Chen et al	2013	≤4,1.05(0.61,1.79); 5,0.95(0.62,1.48); 6, 0.79(0.54,1.16); 7,1.0(reference); 8, 1.36(0.92,2.01); ≥9, 2.36(1.46,3.8)	Sex, age, education, marital status, living status, depression, body mass index, insomnia, hypnotics use, total sleep time, excessive daytime sleepiness, pain, smoking, alcohol drinking, snorers, diabetes mellitus, hypertension, cardiovascular disease, stroke, and gouty arthritis.	CVD (259)
Westerlu-nd et al	2013	≤5, 1.19 (0.92-1.55); 6, 1.05 (0.88-1.25); 7, 1.0 (reference); ≥8, 1.19 (1.00-1.41)	Age, sex, education, employment status, smoking, alcohol, snoring, work schedule, depressive symptoms, self-rated health, physical activity, BMI, diabetes, lipid disturbance, and hypertension	MI (1,908)
Sands-Lincoln et al	2013	≤5, 1.25 (1.13-1.37); 6, 1.07 (1.00-1.14); 7-8, 1.0 (reference); 9, 1.01 (0.88-1.16); ≥10, 1.43 (1.03-1.99)	Age, race, education, income, smoking, BMI, physical activity, alcohol intake, depression, diabetes, high blood pressure, hyperlipidemia, comorbid conditions	CHD (5,359)

Kakizaki et al	2013	≤6, 1.38 (1.02-1.86); 7, 1.0 (reference); 8, 1.36 (1.06-1.73); 9, 1.49 (1.10-2.20); ≥10, 1.41 (1.04-1.92)	Age, sex, total caloric intake, BMI, marital status, education, job, smoking, alcohol, walking, history of cancer, stroke, hypertension, diabetes mellitus, perceived mental stress, self-rated health, physical function	IHD mortality （561）
Hale et al	2013	≤5, 1.09 (0.63-1.89); 6, 0.66 (0.42-1.04); 7-8, 1.0 (reference); ≥9, 1.88 (0.92-3.83)	Age, ethnicity, education, income, fibrinogen, body mass index, low physical exercise, high alcohol intake, ever smoke, elevated blood pressure, diabetes, depression, general health, life satisfaction scale	CHD (132)
Garde et al	2013	<6, 1.46 (1.07-2.00); 6-7, 1.0 (reference); ≥8, 1.2, (0.97-1.49)	Age, BMI, systolic BP, diastolic BP, diabetes, hypertension, physical fitness, alcohol use, smoking, leisure-time physical activity, and social class	IHD (587)
Kim at al	2013	male: 5, 1.21 (1.04-1.42); 6, 0.96 (0.85-1.08); 7, 1.0(reference); 8, 1.00 (0.89-1.12); 9,1.16 (1.00-1.34) female:5, 1.18 (0.98-1.42); 6, 1.13 (0.97-1.31); 7, 1.0(reference); 8, 1.12 (0.96-1.29); 9,1.23 (1.02-1.49)	5-year age groups at cohort entry, sex, ethnicity, education, marital status, history of hypertension or diabetes at enrollment, alcohol consumption, energy intake, body mass index, physical activity, hours spent daily watching television, and smoking history by inclusion of the following variables: [smoking status, average number of cigarettes, average number of cigarettes squared, number of years smoked (time dependent), number of years since quitting (time dependent), and interactions between ethnicity and the smoking variables].	CHD (M:2,096; F:1,380)
Kim at al	2013	Male: ≤5, 1.24 (0.94-1.64); 6, 0.92 (0.74-1.15); 7, 1.0(reference); 8, 0.98 (0.80-1.20); ≥9, 1.16 (0.89-1.50) female: ≤5, 1.18 (0.87-1.59); 6, 1.23 (0.96-1.56); 7, 1.0(reference); 8, 1.10 (0.86-1.40); ≥9,1.29 (0.94-1.75)	5-year age groups at cohort entry, sex, ethnicity, education, marital status, history of hypertension or diabetes at enrollment, alcohol consumption, energy intake, body mass index, physical activity, hours spent daily watching television, and smoking history by inclusion of the following variables: [smoking status, average number of cigarettes, average number of cigarettes squared, number of years smoked (time dependent), number of years since quitting (time dependent), and interactions between ethnicity and the smoking variables].	MI (M:667; F:521)
von Ruesten et al	2012	<6, 1.44 (0.85-2.43); 6-<7, 0.80 (0.53-1.20); 7-<8, 1.0 (reference); 8-<9, 0.82 (0.56-1.19); ≥9, 0.89 (0.54-1.49)	Age, sex, sleeping disorders, smoking, alcohol, walking, cycling, sports, employment status, education, BMI, waist-to-hip ratio, prevalent hypertension at baseline, hypercholesterolemia, coffee, tea, satisfaction with life, satisfaction with health, and intake of antidepressants	MI (197)
Kronholm et al	2011	M:<5,1.2(0.96,1.5); 6, 1.12(0.96,1.31); 7-8, 1.0(reference); 9, 0.95(0.8,1.14); >10,1.27(0.94,1.75); F:<5,1.33 (1.06-1.67); 6, 1.20 (1.01-1.42); 7-8, 1.0(reference); 9, 1.20 (1.00 -1.45); >10, 1.76 (1.34-2.32)	Age, smoking, BMI, systolic blood pressure and total cholesterol	CVD (3,174)
Hoevenaar-Blom et al	2011	≤6, 1.11(0.97,1.27); 7,1.0(reference); 8, 0.95(0.84,1.08);≥9, 0.96(0.77,1.18)	Age, sex, smoking, alcohol, coffee, subjective health, educational level, BMI, total-/HDL cholesterol ratio, systolic blood pressure, CVD risk factor medication, and prevalence of type 2 diabetes	CVD (1,486)
Hamazaki et al	2011	<6,3.49(1.3,9.4); 6-6.9, 1.11(0.55,2.25); 7-7.9,1.0(reference); ≥8, 1.71(0.9,3.24)	Age, type of job, working hours, mental workload, body mass index, mean blood pressure, HbA1c, total cholesterol, current smoking habit, drinking habit, leisure–time physical activity, medication for hypertension, diabetes, and hypercholesterolemia	CVD (64)
Hoevenaar-Blom at al	2011	≤6, 1.19 (1.00-1.40); 7, 1.0 (reference); 8, 0.85 (0.73-1.00); ≥9, 0.78 (0.58-1.04)	Age, sex, smoking, alcohol, coffee, subjective health, educational level, BMI, total-/HDL cholesterol ratio, systolic blood pressure, CVD risk factor medication and prevalence of type 2 diabetes	CHD (1,148)

Chien et al	2010	≤5,0.94(0.65,1.35); 6, 0.91(0.67,1.24); 7,1.0(reference); 8, 1.05(0.8,1.39); ≥9, 1.12(0.81,1.55)	Age, sex, BMI, smoking, current alcohol drinking, marital status, education level, occupation, regular exercise, family history of coronary heart disease, baseline hypertension, diabetes, cholesterol, HDL, triglyceride, glucose, and uric acid level	CVD (420)
Amagai et al	2010	M :<5.9,2.14 (1.11- 4.13); 6.0–6.9,1.04 (0.61-1.76); 7.0–7.9, 1.0(reference); 8.0–8.9, 0.98 (0.69 -1.40); 9.0, 1.33 (0.93-1.92) F: <5.9,1.46 (0.70- 3.04); 6.0–6.9,0.64 (0.38- 1.10); 7.0–7.9, 1.0(reference); 8.0–8.9, 0.85 (0.60 - 1.20); 9.0, 1.28 (0.88-1.87)	Age, systolic blood pressure, total cholesterol, body mass index, smoking habits, and alcohol drinking habits	CVD (481)
Chandola et al	2010	≤5, 1.05 (0.92-1.20); 6, 0.98 (0.83-1.16); 7, 1.0 (reference); 8, 0.99 (0.77-1.27)	Age, sex, ethnicity, employment grade, car access, housing tenure, self-rated health status, total cholesterol, hypertension, BMI, diabetes, smoking, alcohol, exercise, fruit and vegetable consumption	CHD (1,205)
Amagai et al	2010	male: ≤5.9, 1.78 (0.50-6.28); 6.0-6.9, 0.77 (0.25-2.33); 7.0-7.9, 1.0 (reference); 8.0-8.9, 0.69 (0.34-1.41); ≥9, 0.99 (0.47-2.06) female: ≤5.9, 4.93 (1.31-18.61); 6.0-6.9, 0.59 (0.13-2.73); 7.0-7.9, 1.0 (reference); 8.0-8.9, 0.59 (0.21-1.66); ≥9, 0.84 (0.27-2.62)	Age, systolic blood pressure, total cholesterol, body mass index, smoking habits, and alcohol drinking habits	MI (M: 55; F:25)
Suzuki et al	2009	≤5, 1.1(0.62,1.93); 6, 0.85(0.5,1.45); 7,1.0(reference); 8, 1.52(1.01,2.29); 9, 1.55(0.91,2.63); ≥10, 1.95(1.18,3.21)	Age, sex (only in the models for all participants), body mass index, smoking status, alcohol consumption, the frequency of physical activity, socioeconomic status, and mental health, hypertension and diabetes mellitus	CVD (310)
Stone et al	2009	<6, 1.03(0.8-1.31); 6-8, 1.0(reference); >8, 1.21 (0.92–1.61)	Age, body mass index, history of at least one medical condition including diabetes mellitus, Parkinson’s disease, dementia, chronic obstructive pulmonary disease, none skin cancer, and osteoarthritis, history of cardiovascular disease, history of hypertension, walks for exercise, alcohol use, smoking status, depression, cognitive impairment, estrogen use, and benzodiazepine use	CVD (723)
Ikehara et al	2009	M: ≤4, 1.11(0.67-1.83); 5, 0.99 (0.77-1.27); 6, 1.01(0.87-1.18); 7,1.0(reference); 8, 1.11 (1.00-1.24); 9, 1.14 (0.99-1.32); ≥10, 1.56 (1.33-1.83); F: ≤4, 1.28 (0.88-1.86); 5, 1.22 (1.00-1.50); 6, 1.00 (0.86-1.16); 7,1.0(reference); 8, 1.28 (1.14-1.44); 9, 1.37 (1.17-1.62); ≥10, 1.54 (1.28-1.86)	Age, body mass index (quintiles), history of hypertension, history of diabetes, alcohol consumption, smoking, education level, hours of exercise, hours of walking, regular employment, perceived mental stress, depressive symptoms and frequency of fresh fish intake	CVD (4,287)

Ikehara et al	2009	Male: ≤4, 0.29 (0.04-2.05); 5, 1.02 (0.62-1.70); 6, 0.86 (0.63-1.19); 7, 1.0 (reference); 8, 1.02 (0.82-1.27); 9, 0.96 (0.70-1.31); ≥10, 1.12 (0.77-1.63) Female: ≥4, 2.32 (1.19-4.50); 5, 1.64 (1.07-2.53); 6, 1.23 (0.88-1.72); 7, 1.0 (reference); 8, 1.24 (0.94-1.64); 9, 1.52 (1.05-2.19); ≤10, 1.04 (0.63-1.72)	Age, body mass index (quintiles), history of hypertension, history of diabetes, alcohol consumption, smoking, education level, hours of exercise, hours of walking, regular employment, perceived mental stress, depressive symptoms and frequency of fresh fish intake	CHD mortality (M:508; F:373)
Shankar et al	2008	M: ≤5, 1.70 (1.35-2.15); 6, 1.20 (0.99-1.45); 7, 1.0 (reference); 8, 1.10 (0.92-1.32); ≥9, 1.88 (1.48-2.40); F: ≤5, 1.43 (1.09-1.88); 6, 1.04 (0.82-1.31); 7,1.0 (reference); 8, 1.15 (0.92-1.44); ≥9, 1.67 (1.24-2.27)	Age, sex, dialect group, education, year of recruitment, body mass index, smoking, alcohol intake, moderate physical activity, dietary intakes of total calories, fruits, vegetables, fiber, total fat, cholesterol, weekly use of vitamin/mineral supplements, and among women, menopausal status, ever use of postmenopausal hormone replacement therapy	CHD mortality (M:846; F:570)
Lan et al	2007	M: <7, 0.91(0.53-1.57); 7-7.9, 1.0(reference); 8-8.9, 1.4(0.93-2.1); 9-9.9, 1.26(0.8-1.98); ≥10, 1.81(1.13-2.89) F: <7, 1.07 (0.54-2.15); 7-7.9, 1.0(reference); 8-8.9, 1.77 (1.05-2.98); 9-9.9, 1.75 (1.00-3.07); ≥10, 1.85 (1.04-3.27)	Age at 1993, marital status, monthly income, cigarettes smoking, alcohol consumption, body mass index, exercise, disease history (heart disease, stroke, and cancer), depression, afternoon nap duration	CVD (379)
Meisinger et al	2007	male: ≤5, 1.13 (0.66-1.92); 6, 1.05 (0.71-1.55); 7, 1.22 (0.92-1.61); 8,1.0 (reference); ≥9, 1.07 (0.75-1.53) female: ≤5, 2.98 (1.48-6.03); 6, 1.05 (0.49-2.27); 7, 1.34 (0.75-2.40); 8, 1.0 (reference); ≥9, 1.40 (0.74-2.64)	Age, survey, BMI, education, dyslipidemia, alcohol intake, parental history of MI, physical activity, regular smoking, hypertension, diabetes, and menopause status (only women)	MI (M:295; F:85)
Patel et al	2004	≤5,1.04(0.79-1.35); 6, 1.06(0.91-1.25); 7,1.0(reference); 8, 1.12(1.04,1.21); ≥9, 1.56(1.25,1.96)	Age, smoking status, alcohol consumption, physical activity, depression, history of snoring, body mass index, history of cancer, cardiovascular disease, hypertension, or diabetes, and shift-working history	CVD (1,084)
Ayas et al	2003	≤5, 1.39 (1.05-1.84); 6,1.18 (0.98-1.43); 7, 1.10 (0.92-1.31); 8, 1.0 (reference); ≥9, 1.37 (1.02-1.85)	Age, smoking, body mass index, snoring, alcohol consumption, exercise level, postmenopausal hormone use, depression (from 1992), aspirin use (from 1988), family history of MI, hypercholesterolemia, shift work (from 1988), diabetes mellitus, hypertension	CHD (934)
Mallon et al	2002	male: <6, 0.70 (0.30–1.70); 6–8, 1.0(reference); >8, 2.20 (1.00–4.40) female: <6, 1.20 (0.40-4.20); 6-8, 1.0 (reference); >8, 0.70 (0.10-5.20)	Age	CAD mortality (M:71; F:20)
Qureshi et al	1997	<6, 1.3 (1.0–1.8); 6–8, 1.0(reference); >8, 1.1 (0.8–1.5)	Age, sex, race, BMI, education, smoking, systolic blood pressure, cholesterol, diabetes	CHD (413)

BMI: body mass index; CVD: cardiovascular disease; MI: myocardial infarction; CHD: coronary heart disease; CAD: coronary artery disease; IHD: ischemic heart disease; M: male; F: female.

Table S3 Sleep duration and cerebrovascular disease

First author	Publication year	Sleep duration (h/d) and relative risk (95% CI)	Covariates in fully adjusted model	Endpoints (No. of cases)
Kario et al	2021	<6, 2.47(1.08,5.63); 6-9, 1.0(reference); >9, 1.41(0.64,3.09)	Age, sex, body mass index, current smoking, history of diabetes, statin use, aspirin use, antihypertensive medication use, history of cardiovascular disease, total cholesterol, high-density lipo- protein cholesterol, and office systolic blood pressure	Stroke: 52
Zhao et al	2021	<6, 1.725 (1.026–2.899); 6-8, 1.0(reference); >8, 1.223 (0.816–1.833)	Age, gender, smoking status, race, body mass index, prevalent hypertension and diabetes mellitus, apnea- hypopnea index, benzodiazepine use, alcohol use.	Ischemic Stroke:129
Titova et al	2020	Total stroke: <7, 1.04 (0.99–1.09); 7-9, 1.0(reference); ≥9, 1.12 (1.03–1.22); ischemic stroke: <7, 1.03 (0.96–1.32); 7-9, 1.0(reference); ≥9, 1.14 (1.03–1.24); hemorrhagic stroke: <7, 1.13 (0.98–1.30); 7-9, 1.0(reference); ≥9, 1.09 (0.86–1.39)	Age (underlying time scale), sex (as a stratification variable), education, smoking status and pack-years of smoking, alcohol intake, walking/bicycling, exercise, body mass index, and history of hypertension, hypercholesterolemia, and diabetes mellitus	Total stroke: 8,091 IS：6,041 ; HS：1062
Zhou et al	2020	Total stroke: <6, 1.10 (0.69–1.75); 6-7, 1.15 (0.93–1.43); 7-8, 1.0(reference); 8-9, 1.03 (0.91–1.17); ≥9, 1.23 (1.07–1.41) Ischemic stroke: <6, 0.88 (0.48–1.61); 6-7, 1.09 (0.84–1.41); 7-8, 1.0(reference); 8-9, 1.06 (0.91–1.23); ≥9, 1.27 (1.08–1.49) Hemorrhagic stroke: <6, 1.78 (0.77–4.11); 6-7, 1.42 (0.90–2.26); 7-8, 1.0(reference); 8-9, 0.92 (0.68–1.24); ≥9, 1.20 (0.87–1.65)	Age (continuous), sex, year of recruitment (2008–2010, 2013), body mass index (continuous), education level (primary school or below, middle school, high school or higher), smoking status (current, former, or never), drinking status (current, former, or never), regular exercise (yes or no), sleep quality (good, fair, or poor), midday napping (continuous), sleep duration (continuous), hypertension (yes or no), diabetes mellitus (yes or no), hyperlipidemia (yes or no), and family history of stroke (yes or no). Each group adjusted for the other covariates except itself.	Total stroke: 1,557 ischemic stroke:1,151 hemorrhagic stroke:287
Ye et al	2020	Without MetS: <6, 1.421 (0.669, 3.017); 6-7, 0.669 (0.362, 1.237); 7-8, 1.0(reference); 8-9, 0.799 (0.529, 1.209); >9, 0.883 (0.487, 1.602) With MetS: <6, 2.249 (0.973, 5.195); 6-7, 1.013 (0.503, 2.043); 7-8, 1.0(reference); 8-9, 1.228 (0.759, 1.988); >9, 2.014 (1.184, 3.426)	Age, sex, BMI, SBP, depression, blood glucose, triglycerides and physical activity	Stroke： 250 （with MetS:115 without MetS:135）
Krittanawong et al	2020	<7, 1.45 (1.23−1.70); 7-9, 1.0(reference); >9, 1.81 (1.37−2.34)	Age, gender, BMI, marital status, educational level, physical activity, sedentary activity, current cigarette smoking, depression, blood pressure, lipid profiles, and hemoglobin.	Stroke: 1,157
Fan et al	2020	<7, 1.05 (0.95,1.15); 7-9, 1.0(reference); >9, 1.25 (1.10,1.42)	Age, sex, the Townsend Deprivation Index, ethnic, total physical activity level, smoking status, alcohol consumption, and family history of heart diseases or stroke.	Stroke: 2,650
Nutakor et al	2020	<6, 2.67(1.38–5.18); 6-8, 1.0(reference); >8, 2.49(1.50–4.15)	Gender, residence, age, marital status, education, and income	Stroke: 82

Li et al	2020	<6, 1.20 (0.72–2.00); 6-8,1.0(reference); ≥8, 1.79 (1.22–2.63)	Age, sex, continuous positive airway pressure adherence, disease type (cardiac or cerebral), baseline snore frequency, Epworth sleepiness scale score, Hospital Anxiety Depression Scale depression subscale score, and apnea–hypopnea index	Stroke:135
Ji et al	2020	<6, 1.63 (1.23–2.11); 6-8, 1.0(reference); >8, 1.40 (1.08–1.75)	Age, gender, marital status, current employment status, educational level, body mass index, cigarette use, alcohol intake, physical activity, type 2 diabetes mellitus, dyslipidemia, hypertension, heart disease, family history of type 2 diabetes mellitus, family history of hypertension, family history of stroke, fasting plasma sugar, and lipids	Stroke: 617
Bochkarev et al	2019	≤6, 0.9 (0.7; 1.2); 6-7,0.9 (0.7; 1.1); 7-8, 1.0(reference); 8-9, 1.2 (0.8; 1.8); >9, 0.8 (0.5; 1.4)	Sex, age, body mass index, office blood pressure, smoking and low physical activity	Stroke: 422
Petrov et al	2018	Black race: <6.0, 0.49(0.28–0.85); 6.0–6.9,1.01 (0.71–1.44); 7.0–8.9, 1.00 (reference); >9, 1.10 (0.50–2.07); White race: <6.0, 1.40 (0.91–2.13); 6.0–6.9,1.01 (0.74–1.39); 7.0–8.9, 1.00 (reference); >9, 1.19 (0.79–1.81)	Age, sex, race, education, income, 4-item Center for Epidemiologic Studies–Depression questionnaire score, smoking status, history of atrial fibrillation, heart disease, left ventricular hypertrophy, diabetes, and sleep-disordered breathing status based on the Berlin Questionnaire.	Stroke:460<br>Black:172<br>White:288
Kawachi et al	2016	Total stroke: ≤6, 0.77 (0.59–1.01); 7, 1.0(reference); 8, 1.13 (0.91–1.40); ≥9, 1.51 (1.16–1.97); Ischemic stroke: ≤6, 0.93 (0.64–1.34); 7, 1.0(reference); 8, 1.33 (0.99–1.80); ≥9, 1.65 (1.16–2.35); Hemorrhagic stroke: ≤6, 0.64 (0.42–0.98); 7, 1.0(reference); 8, 0.90 (0.64–1.26); ≥9, 0.96 (0.60–1.54)	Sex, age, education years, marital status, histories of hypertension and diabetes, body mass index, physical activity score, smoking status, and alcohol consumption	Total stroke: 611 M:296; F: 315; IS: 354; HS:217
Song et al	2016	total stroke:<6, 0.92 (0.80-1.05); 6-8, 1.0 (reference); >8, 1.29 (1.01-1.65); Ischemic stroke: <6, 0.89 (0.77–1.03); 6-8, 1.0 (reference); >8, 1.19 (0.90–1.57); Hemorrhagic stroke: <6, 1.03 (0.77–1.37); 6-8, 1.0 (reference); >8, 1.67 (1.01–2.75)	Age, sex, marital status, family per member monthly income, education level, smoking status, drinking status, physical activity, family history of stroke, body mass index, systolic blood pressure, diastolic blood pressure, fasting blood glucose, total cholesterol, hypotensive drug use, lipid-lowering drug use, hypoglycemic drug use, history of myocardial infarction, and snoring status, sensitive C-reactive protein, and atrial fibrillation	Total stroke:3,135; IS: 2,504; HS:631
Smagula et al	2016	<6, 1.09 (0.77–1.55); 6-8, 1.0(reference); >8, 1.68 (1.39–2.02)	Age, physical activity and history of chronic diseases	Stroke: 535
Cai et al	2015	4-5, 0.91 (0.70–1.18); 6, 0.99 (0.79–1.23); 7 1.0(reference); 8, 1.28 (1.04–1.58); 9, 1.31 (0.94–1.82); ≥10, 2.35 (1.78–3.09)	Age, education, income, smoking, alcohol consumption, tea consumption, comorbidity score, history of night-shift work, participation in regular exercise, body mass index, and waist-to-hip ratio, cardiovascular disease, upper gastrointestinal tract	Stroke: 746
Helbig et al	2015	Male: ≤5, 1.36 (0.95–1.94); 6, 0.92 (0.70–1.22); 7-8, 1.0(reference); 9, 1.05 (0.78–1.43); ≥10, 1.38 (0.98–1.94) Female: ≤5, 0.68 (0.40–1.18); 6, 1.25 (0.91–1.70); 7-8, 1.0(reference); 9, 1.09 (0.76–1.57); ≥10, 0.91 (0.55–1.51)	Age, survey, education, physical activity, alcohol consumption, current smoking, dyslipidemia activity, BMI, hypertension, diabetes	Stroke:<br>Male: 508; Female: 318

Leng et al	2015	All: <6, 1.18 (0.91, 1.53); 6-8, 1.0(reference); >8,1.46 (1.08, 1.98) Male:<6, 1.08 (0.75-1.57); 6-8, 1.0(reference); >8 1.21 (0.80-1.82) Female: <6, 1.25 (0.86-1.83); 6-8, 1.0(reference); >8 1.80 (1.13-2.85)	Age, sex, social class, education, marital status, smoking, alcohol intake, hypnotic drug use, family history of stroke, body mass index, physical activity, depression, hypnotic drug use, systolic blood pressure, diastolic blood pressure, preexisting diabetes and myocardial infarction, cholesterol level, and hypertension drug use	Stroke: 346; Male: 198; Female: 148
Pan et al	2014	Total stroke: ≤5, 1.25 (1.05–1.50); 6, 1.01 (0.87–1.18); 7, 1.0(reference); 8, 1.09 (0.95–1.26); ≥9, 1.54 (1.28–1.85) Hemorrhagic stroke: ≤5, 1.37 (1.12–1.68); 6, 1.04 (0.87–1.24); 7, 1.0(reference); 8, 1.14 (0.96–1.34); ≥9, 1.68 (1.36–2.06) Hemorrhagic stroke: ≤5, 0.92 (0.62–1.36); 6, 0.91 (0.67–1.24); 7, 1.0(reference); 8, 0.97 (0.73–1.29); ≥9, 1.14 (0.76–1.72)	Age, year of recruitment, sex, dialect, education, body mass index, alcohol drinking, years of smoking, dose of smoking, moderate activity, energy intake, dietary intakes of vegetables, fruits, fiber, polyunsaturated fatty acids, self-reported history of physician diagnosed hypertension, diabetes, stroke and coronary heart disease, and history of cancer reported by the nationwide cancer registry	Total stroke: 1,381; hemorrhagic stroke: 322; ischemic stroke: 1,059
Ruiter Petrov et al	2014	< 6, 1.43(0.88-2.32); 6-6.9, 1.16(0.79-1.69); 7-7.9, 1.00 (reference); 8-8.9, 1.17(0.84-1.62); 9, 1.44(0.86-2.42)	Age, race, sex, income, education, region	Stroke: 224
Kakizaki et al	2013	≤6, 1.05 (0.84–1.30); 7, 1.0(reference); 8, 1.17 (0.99–1.39); 9, 1.30 (1.06–1.60); ≥10, 1.51 (1.24–1.85)	Age; sex; total caloric intake; body mass index in $kg*m^{-2}$; marital status; level of education; job status; history of myocardial infarction; history of cancer; history of stroke; history of hypertension; history of diabetes mellitus; smoking status; alcohol drinking; time spent walking; perceived mental stress; self-rated health, physical function.	Stroke: 1,165
Kim at al	2013	Male: ≤5, 1.02 (0.74-1.40); 6, 1.10 (0.88-1.37); 7, 1.0(reference); 8, 1.13 (0.91-1.39); ≥9, 1.35 (1.03-1.75) Female: ≤5, 1.16 (0.88-1.52); 6, 0.99 (0.79-1.23); 7, 1.0(reference); 8, 1.07 (0.87-1.33); ≥9, 1.39 (1.06-1.83)	5-year age groups at cohort entry, sex, ethnicity, education, marital status, history of hypertension or diabetes at enrollment, alcohol consumption, energy intake, body mass index, physical activity, hours spent daily watching television, and smoking history by inclusion of the following variables: [smoking status, average number of cigarettes, average number of cigarettes squared, number of years smoked (time dependent), number of years since quitting (time dependent), and interactions between ethnicity and the smoking variables]	Stroke: 1,259; male：632; female：627
Westerlund et al	2013	≤5, 1.05 (0.80–1.37); 6, 0.95 (0.79–1.14); 7, 1.00 (Reference); ≥8, 0.87 (0.72–1.04)	Age, sex, education, employment status, smoking, alcohol, snoring, work schedule, depressive symptoms, self- rated health, physical activity, BMI, diabetes, lipid disturbance, and hypertension	Stroke: 1,685
von Ruesten et al	2012	<6, 2.06 (1.18–3.59); 6-7, 1.13 (0.72–1.77); 7-8, 1.0(reference); 8-9, 1.16 (0.77–1.73); ≥9,1.65 (1.00–2.73)	age, sex, sleeping disorders, alcohol intake from beverages, smoking status, walking, cycling, sports, employment status, and education, potential mediators: BMI (kg/m2), waist-to-hip ratio, prevalent hypertension at baseline (yes/no), and history of high blood lipid levels at baseline, consumption of caffeinated beverages, satisfaction with life, satisfaction with health, and intake of antidepressants.	Stroke:169

Hamazaki et al	2011	<6, 1.84 (0.23-14.90); 6-6.9, 0.96 (0.30 to 3.10); 7-7.9, 1.0(reference); ≥8, 2.25 (0.91-5.57)	Age, sex, education, employment status, smoking, alcohol, snoring, work schedule, depressive symptoms, selfrated health, physical activity, BMI, diabetes, lipid disturbance, and hypertension	Stroke: 30
Amagai et al	2010	Male: <5.9, 2.00 (0.93–4.31); 6.0–6.9, 1.13 (0.63–2.03); 7.0–7.9, 1.00 (reference); 8.0–8.9, 1.03 (0.69–1.53); 9.0, 1.39 (0.92–2.10) Female: <5.9, 0.97 (0.39–2.41); 6.0–6.9, 0.68 (0.39–1.18); 7.0–7.9, 1.00 (reference); 8.0–8.9, 0.86 (0.60–1.23); 9.0, 1.29 (0.86–1.91)	Age, systolic blood pressure, total cholesterol, body mass index, smoking habits, and alcohol drinking habits.	Stroke: 411; male:207; female: 204
Ikehara et al	2009	Male: Total stroke: ≤4, 1.56 (0.82-2.94); 5, 0.85 (0.58-1.26); 6, 0.95 (0.76-1.20); 7, 1.0(reference); 8, 1.11 (0.95-1.30); 9, 1.14 (0.92-1.42); ≥10, 1.66 (1.31-2.08) ; Hemorrhagic stroke:≤4, 2.15 (0.78-5.89); 5, 1.20 (0.64-2.26); 6, 1.13 (0.77-1.65); 7, 1.0(reference); 8, 1.27 (0.97-1.66); 9, 1.01 (0.66-1.53); ≥10, 1.56 (0.99-2.45); Ischemic stroke: ≤4, 1.28 (0.52-3.15); 5, 0.70 (0.41-1.20); 6, 0.76 (0.55-1.04); 7, 1.0(reference); 8, 1.02 (0.83-1.26); 9, 1.18 (0.90-1.55); ≥10, 1.58 (1.19-2.12) ; Female: Total stroke: ≤4, 1.07 (0.59-1.91); 5, 0.99 (0.72-1.37); 6, 0.93 (0.75-1.16); 7, 1.0(reference); 8, 1.24 (1.05-1.47); 9, 1.29 (1.01-1.64); ≥10, 1.69 (1.29-2.20); Hemorrhagic stroke:≤4, 0.68 (0.22-2.15); 5, 0.93 (0.57-1.52); 6, 0.82 (0.60-1.14); 7, 1.0(reference); 8, 1.17 (0.91-1.51); 9, 1.16 (0.78-1.72); ≥10, 0.78 (0.43-1.40); Ischemic stroke: ≤4, 1.57 (0.79-3.13); 5, 1.26 (0.82-1.94); 6, 1.10 (0.79-1.51); 7, 1.0(reference); 8, 1.29 (1.00-1.67); 9, 1.38 (0.98-1.95); ≥10, 2.37 (1.70-3.32)	Age, body mass index (quintiles), history of hypertension, history of diabetes, alcohol consumption, smoking, education level, hours of exercise, hours of walking, regular employment, perceived mental stress, depressive symptoms and frequency of fresh fish intake.	Male: total stroke: 2,038; hemorrhagic stroke: 339; ischemic stroke: 607 Female: total stroke: 926; hemorrhagic stroke: 379; ischemic stroke: 464
Chen et al	2008	≤6, 1.14 (0.97 -1.33); 7 1.0(reference); 8, 1.24(1.04-1.47); ≥9, 1.70 (1.32-2.21)	Age, sex, total caloric intake, body mass index in, marital status, level of education, job status, history of myocardial infarction, history of cancer, history of stroke, history of hypertension, history of diabetes mellitus, smoking status, alcohol drinking, time spent walking, perceived mental stress, self-rated health, physical function	Stroke 1,166
Qureshi et al	1997	<6, 1.0 (0.7-1.5); 6-8, 1.0(reference); >8, 1.5 (1.1-2.0)	Age, body mass index, systolic blood pressure, diastolic blood press, smoking status, drinking habits and physical activity	Stroke:285

IS: Ischemic stroke; HS: Hemorrhagic stroke

Table S4 Comparison between previous meta-analysis and the present study. Cardiovascular disease (A); Cerebrovascular disease (B).

(A)

Author&Year	Search endpoint	Exposure	Outcome	Studies included	Publication bias	DR analysis method	Assessment of risk bias (NOS)	Directness
Jiawei Yin, 2017	2016-12	Sleep duration	CVD&CHD	CVD:26 cohort studies CHD:22 cohort studies	Existence	Greenland and Longnecker	Study quality is moderate to excellent	Mixed exposure and outcome
**This study**	**2022-6**	**Nighttime sleep & 24-hour sleep duration**	**CVD&CHD&MI**	**CVD:32 cohort studies CHD&MI: 31 cohort studies**	**No publication bias**	**One stage REMR model can included more studies***	**Study quality is deemed moderate, possibly because we adopted more rigorous rating criteria**	**Added a specific outcome classification**

(B)

Author&Year	Search endpoint	Exposure	Outcome	Studies included	Publication bias	DR analysis method	Risk of bias (NOS)	Directness
Wenzhen Li, 2016	2016-2	Sleep duration	Stroke	Stroke:16 cohort studies	Existence	Greenland and Longnecker	Study quality is moderate to excellent	Mixed exposure and outcome
**This study**	**2022-6**	**Nighttime sleep & 24-hour sleep duration**	**Total stroke、Ischemic stroke and Hemorrhagic stroke**	**Total stroke: 32 cohort studies; Ischemic & Hemorrhagic stroke: 6 cohort studies**	**No publication bias**	**One stage REMR model can included more studies***	**Study quality is deemed moderate, possibly because we adopted more rigorous rating criteria**	**Added a specific outcome classification**

* One stage REMR model can included studies with only two types of relative risk (RR) and 95% confidence interval (CI) for sleep duration.
